# Comparative analysis of the mitochondrial genomes of four *Dendrobium* species (Orchidaceae) reveals heterogeneity in structure, synteny, intercellular gene transfer, and RNA editing

**DOI:** 10.3389/fpls.2024.1429545

**Published:** 2024-07-30

**Authors:** Le Wang, Xue Liu, Yongde Wang, Xingjia Ming, Junsheng Qi, Yiquan Zhou

**Affiliations:** ^1^ Chongqing Key Laboratory of Special Chinese Materia Medica Resources Utilization and Evaluation, Endangered Medicinal Breeding National Engineering Laboratory, Chongqing Academy of Chinese Materia Medica, Chongqing, China; ^2^ College of Life Science and Food Engineering, Chongqing Three Gorges University, Chongqing, China; ^3^ Daba Mountain Medical Animals and Plants of Chongqing Observation and Research Station, Chongqing Academy of Chinese Materia Medicinal, Chongqing, China

**Keywords:** *Dendrobium* species, mitochondrial genome, chloroplast genome, homologous sequence, RNA editing

## Abstract

The genus *Dendrobium*, part of the Orchidaceae family, encompasses species of significant medicinal, nutritional, and economic value. However, many *Dendrobium* species are threatened by environmental stresses, low seed germination rates, and overharvesting. Mitochondria generate the energy necessary for various plant life activities. Despite their importance, research on the mitochondrial genomes of *Dendrobium* species is currently limited. To address this gap, we performed a comprehensive genetic analysis of four *Dendrobium* species—*D. flexicaule*, *D. nobile*, *D. officinale*, and *D. huoshanense*—focusing on their mitochondrial and chloroplast genomes to elucidate their genetic architecture and support conservation efforts. We utilized advanced sequencing technologies, including Illumina for high-throughput sequencing and Nanopore for long-read sequencing capabilities. Our findings revealed the multichromosomal mitochondrial genome structures, with total lengths ranging from 596,506 bp to 772,523 bp. The mitochondrial genomes contained 265 functional genes, including 64-69 protein-coding genes, 23-28 tRNA genes, and 3 rRNA genes. We identified 647 simple sequence repeats (SSRs) and 352 tandem repeats, along with 440 instances of plastid-to-mitochondrial gene transfer. Additionally, we predicted 2,023 RNA editing sites within the mitochondrial protein-coding genes, predominantly characterized by cytosine-to-thymine transitions. Comparative analysis of mitochondrial DNA across the species highlighted 25 conserved genes, with evidence of positive selection in five genes: *ccmFC*, *matR*, *mttB*, *rps2*, and *rps10*. Phylogenetic assessments suggested a close sister relationship between *D. nobile* and *D. huoshanense*, and a similar proximity between *D. officinale* and *D. flexicaule.* This comprehensive genomic study provides a critical foundation for further exploration into the genetic mechanisms and biodiversity of *Dendrobium* species, contributing valuable insights for their conservation and sustainable utilization.

## 1 Introduction

Plastids and mitochondria are essential organelles in plant cells, each containing a genome distinct from the nuclear genome. Plastids play a crucial role in photosynthesis and various biosynthetic processes, while mitochondria are the primary sites for oxidative phosphorylation and ATP synthesis, both vital for plant growth and development (Liu et al., 2023; Lian et al., 2024; Wang et al., 2024). The plastid genome is maternally inherited, rich in gene content, structurally conserved, and evolves relatively slowly. These characteristics make plastid genomes valuable for phylogenetic studies, biological research, and examining the degradation of photosynthetic genes in plants. In contrast, plant mitochondrial genomes (mtDNA) have traditionally garnered less attention than plastid or nuclear genomes due to their highly variable structures and repetitive DNA content, which present significant challenges for comprehensive genomic sequencing (Xiong et al., 2021; Han et al., 2022). Meanwhile, variations in mtDNA size are largely due to differences in the amount of non-coding content, which originates from diverse sources. These sources include repeats, large duplications (Maréchal and Brisson, 2010), intracellular gene transfers (IGT) from nuclear or plastid genomes (Gandini et al., 2019), and horizontal gene transfers (HGT). Notably, RNA editing events are considered a general feature of plant mtDNAs (Varré et al., 2019). Particularly, Cytosine (C)-to-Uracil (U) RNA editing occurs at approximately 200-800 mRNA sites (Mower, 2008; Sloan et al., 2010; Rice et al., 2013; Richardson et al., 2013). Additionally, 92% of RNA editing sites result in changes to amino acid sequences. For example, hydrophilic amino acids are often converted into hydrophobic ones, which can enhance protein folding and gene functionality (Giegé and Brennicke, 1999). The study of mitochondrial genomes in angiosperms not only elucidates phylogenetic relationships between species but also facilitates the investigation of intraspecific differentiation.

To date, it is known that the majority of plant mitochondrial genomes exhibit a single circular chromosome structure, but this is not universal (Qu et al., 2024). For example, the mitochondrial genomes of *Lilium tsingtauense* (Qu et al., 2024), *Populus simonii* (Bi et al., 2022), *Amborella trichopoda*, *Gastrodia elata* (Yuan et al., 2018), *Populus deltoides* (Qu et al., 2023), *Cucumis sativus*, wheat and rape exhibit complex multichromosomal structures (Alverson et al., 2011; Yu et al., 2022). Interestingly, the largest circular chromosome (1,556 kb) in the *C. sativus* mtDNA has greater protein-coding capability than the other two smaller chromosomes (45 and 84 kb). This suggests that the three mitochondrial chromosomes of *C. sativus* replicate independently (Alverson et al., 2011; Wu et al., 2022). Similarly, the mitochondrial genomes of *Lophophytum mirabile* and *Silene noctiflora* consist of 54 and 128 distinct circular chromosomes, respectively, highlighting the extreme complexity of plant mitochondrial structures (Bi et al., 2022). Currently, the *D. officinale* and *D. huoshanense* mtDNAs were found to have 22 and 19 chromosomes, respectively. This indicated that the differently length isoforms during rapid evolution of *Dendrobium* mtDNAs structure might be caused by repeat-mediated rearrangement events (Wang et al., 2023). Exploring mitochondrial genome differences among species or subspecies of endangered plants elucidates the role of mitochondria in biological evolution. This understanding can significantly enhance applications in genetic breeding, conservation, and research.

*Dendrobium*, the second-largest genus in the Orchidaceae family, encompasses approximately 80 species, many of which hold significant medicinal, nutritional, ornamental, and economic value (Xu et al., 2022). However, extreme ecological conditions and insufficient protective measures for wild *Dendrobium* resources, several species, notably *D. huoshanense* and *D. flexicaule*, are considered rare and endangered. Due to the significant morphological similarities among *D. flexicaule*, *D. nobile*, *D. officinale*, and *D. huoshanense*, we sequenced the mitochondrial and plastid genomes of four important medicinal *Dendrobium* species using both Illumina and Nanopore sequencing technologies in this study. Furthermore, we analyzed codon usage preference, sequence repeat, Ka/Ks ratio, RNA editing sites and collinearity in the four *Dendrobium* mtDNAs. Additionally, we also analyzed homologous fragments between mitochondria and chloroplasts and phylogenetic analysis. Our analyses provide the theoretical foundation for the identification, conservation, and the development of molecular markers and genetic breeding strategies of *Dendrobium* species.

## 2 Materials and methods

### 2.1 Material collection, genome extraction, and sequencing

Fresh and healthy leaves from four individual plants (*D. flexicaule*, *D. nobile*, *D. officinale*, *D. huoshanense*) were collected and cultivated in the germplasm resources herb garden at the Chongqing Academy of Chinese Materia Medica, Chongqing, China (**Supplementary Table S2.1**). The integrity and concentration of DNA were assessed using 0.75% agarose gel electrophoresis, a NanoDrop One spectrophotometer (Thermo Fisher Scientific), and a Qubit 3.0 Fluorometer (Life Technologies, Carlsbad, CA, USA). DNA samples were sheared using a Covaris ultrasonic disruptor and prepared into 300 bp insert size Illumina sequencing libraries using the Nextera DNA Flex Library Prep Kit (Illumina, San Diego, CA, USA). Sequencing was performed on the Illumina NovaSeq platform (**Supplementary Figure S1**). Raw reads were processed to remove adapters, unknown nucleotides (Ns), and low-quality bases using SOAPnuke v.2.1.4 (https://github.com/BGI-flexlab/SOAPnuke), retaining clean data for further analysis. For Oxford Nanopore sequencing, libraries were prepared using the SQK-LSK109 ligation kit and sequenced on a PromethION sequencer (Oxford Nanopore Technologies, Oxford, UK) for 48-hour runs. Base-calling was conducted using GUPPY software (Shen et al., 2022).

### 2.2 Assembly and annotation of mtDNA and cpDNA

We combined next-generation sequencing (NGS) data from Illumina and third-generation sequencing (TGS) data from Nanopore to assemble the mitochondrial genomes of four *Dendrobium* species. Using GetOrganelle v.1.7.5 (Jin et al., 2020), we assembled the plant mitochondria with default parameters, incorporating both next-generation and third-generation DNA sequencing data to produce a graphical plant mitochondrial genome (Jin et al., 2020). The bandage tool was employed to visualize this mitochondrial genome and manually eliminate extended segments originating from chloroplast and nuclear genomes (Wick et al., 2015). Subsequently, bwa software was used to align the Nanopore data to the graphical mitochondrial genome fragment, which helped resolve repetitive regions within the plant mitochondrial genome (Li and Durbin, 2009). As a result, we obtained the mitochondrial genomes of the four *Dendrobium* species, each with multiple branches.

For annotating the protein-coding genes (PCGs) of the mitochondrial genome, we used reference genomes from the complete mitochondrial genomes of *Dendrobium* (LC704589.1-LC704614.1). The Geseq software program (Tillich et al., 2017) was employed for mitochondrial genome annotation. tRNA-encoding genes were annotated with tRNAscan-SE software (Lowe and Eddy, 1997), and rRNA-encoding genes were annotated using BLASTN software (Chen et al., 2015). Any annotation errors in the mitochondrial genome were manually corrected using Apollo software (Lewis et al., 2002).

### 2.3 Comparison of codon usage bias

The Phylosuite software program was used with default parameters to extract protein-coding sequences from the mitochondrial genomes of four *Dendrobium* species (Zhang et al., 2020). The MEGA v.7.0 software was employed to analyze the coding bias of mitogenomic protein-coding genes (PCGs) and to calculate the relative synonymous codon usage (RSCU) values (Kumar et al., 2016). The online tool CUSP (https://www.bioinformatics.nl/cgi-bin/emboss/cusp) was used to determine the overall GC content, as well as GC1, GC2, and GC3 content in the mtDNA of the four *Dendrobium* species. The CodonW v.1.4.2 software with default settings was used to compute the effective number of codons (ENC) (Sharp and Li, 1986), which measures the diversity of codon usage in a gene, with values typically ranging from 20 (indicating each amino acid uses only one codon) to 61 (indicating equal usage of all codons) (Wright, 1990). The GC content at different positions in the four *Dendrobium* species was visualized using TBtools software with default parameters (Chen C. et al., 2020). Additionally, a scatter plot of the average GC1 and GC2 (GC12) values against GC3 values was created using Microsoft Excel 2021 to analyze neutrality in the four *Dendrobium* species.

### 2.4 Repeat sequences and intracellular gene transfer of the mitochondrial genome

The online tools MISA (https://webblast.ipk-gatersleben.de/misa/), TRF (https://tandem.bu.edu/trf/trf.unix.help.html), and the REPuter network server were utilized for analyzing simple sequence repeats (SSRs), tandem repeats, and dispersed repeats, respectively. The parameters for MISA were set as 1-10, 2-5, 3-4, 4-3, 5-3, and 6-3. For TRF, the parameters were 2 7 7 80 10 50 2000 –f -d -m. REPuter was used with a hamming distance of 3, a maximum of 5,000 computed repeats, and a minimal repeat size of 30. Default parameters were applied for the analysis of dispersed repeats (Benson, 1999; Kurtz et al., 2001; Beier et al., 2017; Shen et al., 2024).

We assembled and annotated the chloroplast genome using Getorganelle software and CPGAVAS2 respectively (Shi et al., 2019; Jin et al., 2020). The Circos package and Excel 2021 were employed to visualize the resulting plots. Additionally, BLASTN software (parameters: e-value=1e−6; word size=7) was used for analyzing homologous fragments, and the Circos package was utilized to visualize intracellular gene transfers (IGT) (Zhang et al., 2013; Chen et al., 2015).

### 2.5 Prediction of RNA editing sites and comparison analysis of collinearity

RNA editing sites were predicted using PREPACT3 (http://www.prepact.de/) with a cutoff value of 0.001 (Lenz et al., 2018). Homologous sequences longer than 500 bp were retained to construct multiple synteny plots as conserved collinear blocks and pairwise comparisons of individual mitochondrial genomes were conducted using BLASTN with the parameters set to e-value of ≤1e−10 and a matching rate of ≥80% (Qu et al., 2023). The MCscanX program was employed to generate multiple synteny plots for the four *Dendrobium* species (Wang et al., 2012).

### 2.6 Phylogenetic tree based on the PCGs

23 species closely related to the four *Dendrobium* species were selected, and their mitochondrial genomes were downloaded from NCBI. *Magnolia biondii* and *Liriodendron tulipifera* were designated as outgroups. Twenty-one conserved protein-coding genes (*atp1*, *atp4*, *atp6*, *ccmB*, *ccmC*, *ccmFC*, *ccmFN*, *cob*, *cox1*, *cox2*, *cox3*, *matR*, *nad1*, *nad2*, *nad3*, *nad4*, *nad5*, *nad6*, *nad7*, *nad9*, and *rps12*) were aligned using the MAFFT software with default parameters (Katoh and Standley, 2013). A phylogenetic tree was constructed using IQ-TREE v.1.6.12 with the parameters “-m MFP -B 1000 –bnni -T AUTO “ and the model “GTR+F+I+I+R2”. The results were visualized using ITOL v.4.0 software (Nguyen et al., 2015; Bi et al., 2022).

## 3 Results

### 3.1 Variation in the structure of mitochondrial and plastid genomes

**Figure 1:**
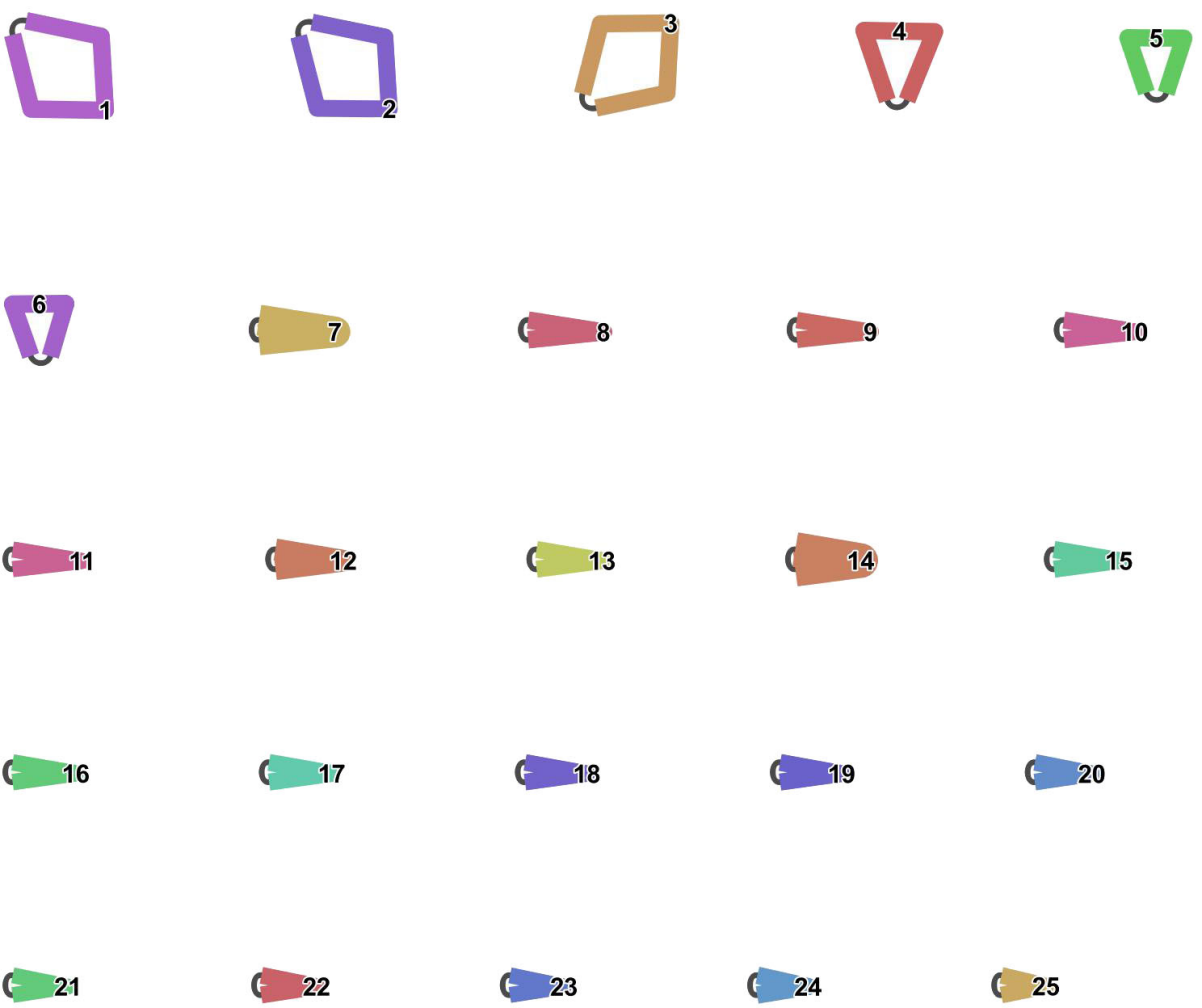
Sketch of the mitochondrial genome of *Dendrobium nobile*. Based on the Nanopore, sketch of the mitogenomes were assembled. Sketches of mitochondrial genomes of *D. huoshanense*, *D. flexicaule* and *D. officinale* were uploaded in **Additional File 1** (**Supplementary Figure S2**).

**Figure 2:**
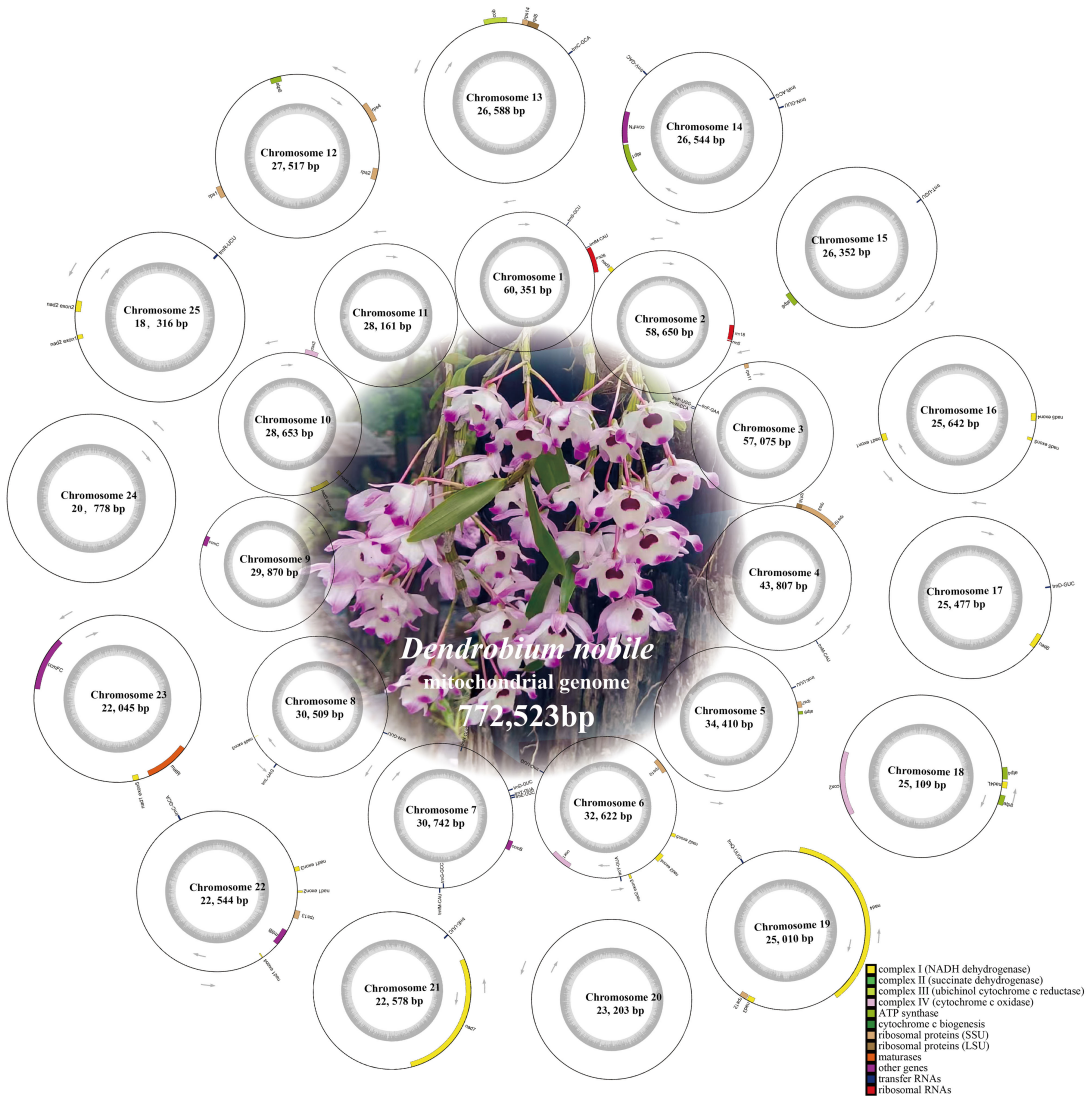
*Dendrobium nobile* mitogenome map. Genes are classified into different categories based on different colors, with the gray area within the circles representing different GC contents. Mitogenome maps of *D. huoshanense*, *D. flexicaule* and *D. offcinale* were uploaded in **Additional File 1** (**Supplementary Figure S3**).

Illumina sequencing generated a total of 47.7 Gb of raw data, with the following breakdown: *D. flexicaule* (11.3 Gb), *D. nobile* (13.2 Gb), *D. huoshanense* (11.5 Gb), and *D. officinale* (11.7 Gb). Nanopore sequencing contributed approximately 45.9 Gb of data across the four species: *D. flexicaule* (11.3 Gb), *D. nobile* (12.9 Gb), *D. huoshanense* (10.5 Gb), and *D. officinale* (11.2 Gb). The mitochondrial and plastidial genomes of these species are available in GenBank (https://www.ncbi.nlm.nih.gov), with corresponding accession numbers provided in **Supplementary Tables S2.2**–**S2.5**. The mitochondrial and plastidial genomes were analyzed to identify variations and conservations within the *Dendrobium* species. Using Bandage, we graphically represented the assembled mtDNAs based on Nanopore data, manually removing nodes from the nuclei and chloroplasts (**Figure 1**). These nodes are independent of each other and capable of self-organizing into circular structures, revealing that the mitochondrial genomes of these species exhibit a complex multibranched conformation with multiple single circular structures (**Figure 2**). Detailed analysis showed that *D. flexicaule*’s mitochondrial DNA comprises 20 circular chromosomes, *D. nobile* has 25, *D. officinale* has 21, and *D. huoshanense* has 20 circular chromosomes. The sizes of these mitochondrial genomes vary significantly: *D. flexicaule* measures 596,506 bp, *D. nobile* 772,523 bp, *D. officinale* 625,267 bp, and *D. huoshanense* 650,957 bp. The GC content was highly similar among the mitochondrial genomes of the four *Dendrobium* species (**Table 1**).

**Table 1:** Features of four *Dendrobium* mitogenomes.

	*D. huoshanense*	*D. nobile*	*D. flexicaule*	*D. officinale*
Accession	OR413847-OR413866	OR413867-OR413891	OR413892-OR413911	OR413912-OR413932
Length	650, 957bp	772, 523bp	596, 506bp	625, 267bp
GC%	43.41%	43.44%	43.54%	43.64%
Genes	69	68	64	64
tRNA	28	27	24	23
rRNA	3	3	3	3
Protein coding genes	37	37	36	37

Among the four *Dendrobium* species, *D. huoshanense* possesses the most genes with a total of 69, followed closely by *D. nobile* with 68, and both *D. flexicaule* and *D. officinale* with 64 genes each (**Supplementary Tables S2.6**–**S2.9**). All species showed significant conservation in the number and types of rRNA genes, though differences were noted in their tRNA-encoding genes and protein-coding genes (PCGs). Specifically, *D. flexicaule* was unique in the loss of the *rps11* gene. Variations in the types and quantities of tRNA-encoding genes were observed among the species, particularly in *trnM-CAU, trnG-GCC, trnN-GUU*, and *trnQ-UUG*. Compared to *D. flexicaule*, *D. huoshanense* had three additional tRNA-encoding genes (*trnL-CAA, trnR-ACG*, and *trnT-UGU*) but lacked *trnH-GUG*. *trnI-CAU* gene was missing in *D. nobile* but additional genes *trnR-ACG* and *trnT-UGU* were present. *D. officinale* lacked *trnH-GUG* but had an additional *trnT-UGU* gene (**Supplementary Table S2.10**).

Chloroplast genomes of four *Dendrobium* species have also been sequenced and assembled. We found that the chloroplast genomes displayed smaller variations in size compared to their mitochondrial counterparts. The lengths of cpDNA were 150,602 bp for *D. nobile*, 150,529 bp for *D. huoshanense*, 152,588 bp for *D. flexicaule*, and 152,213 bp for *D. officinale* (**Supplementary Figure S4**). *D. nobile* and *D. huoshanense* shared similar GC content, as well as comparable tRNA, rRNA, and protein-coding regions (**Table 2**). The results of the mVISTA analysis demonstrated that the chloroplast genomes of the four species are highly similar, especially in the IR and coding regions, with lower differentiation observed in the SC and non-coding areas (**Supplementary Figure S5**). This pattern of conservation and variation provides valuable insights into the evolutionary dynamics and functional adaptations within the genus *Dendrobium*.

**Table 2:** Features of chloroplast genomes of the four *Dendrobium* species based on the Orchidaceae.

Species	Accession number	Length	GC content	LSC	SSC	IRs
*D. huoshanense*	OR387325	150, 529bp	38%	84,792bp	14, 171bp	51, 566bp
*D. nobile*	OR387323	150, 602bp	38%	84, 781bp	13, 799bp	52, 022bp
*D. flexicaule*	OQ360111	152, 588bp	37%	85, 270bp	14, 634bp	52, 684bp
*D. officinale*	OR387324	152, 213bp	37%	85, 146bp	14, 449bp	52, 618bp

### 3.2 Codon usage bias and mitogenomic evolution in *Dendrobium* species

**Figure 3:**
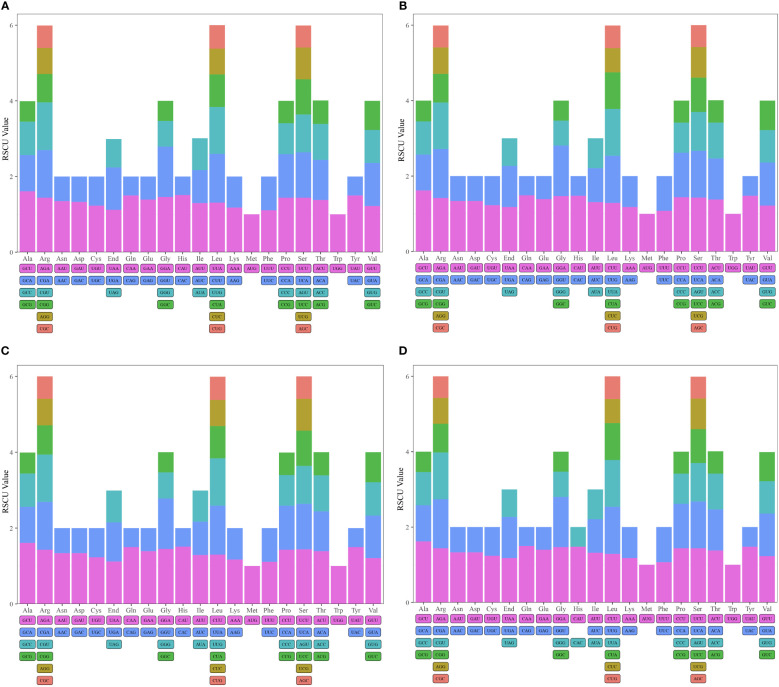
Relative synonumous codon usage (RSCU) in mitogenomes of *D. huoshanense* **(A)**, *D. nobile* **(B)**, *D. flexicaule* **(C)**, *D. officinale* **(D)**. Codon families are indicated on the x-axis. RSCU values are shown on the y-axis.

Codon usage bias is crucial for understanding gene expression and evolutionary patterns. In our analysis, we assessed the codon usage of 36 unique protein-coding genes (PCGs) from *D. flexicaule*, and 37 from each of *D. officinale*, *D. nobile*, and *D. huoshanense*. The results revealed a distinct amino acid bias, with Relative Synonymous Codon Usage (RSCU) values above 1 indicating preferences (**Figure 3**, **Supplementary Tables S2.11**–**S2.14**). The mitochondrial genomes showed a general codon bias; for instance, Ala favored GCU, with RSCU values around 1.62 in *D. officinale* and *D. huoshanense*, and 1.61 in both *D. flexicaule* and *D. nobile*. Gln showed a preference for CAA in *D. huoshanense* and *D. officinale*, while His in *D. flexicaule* and *D. nobile* had an RSCU of 1.51. Notably, Lys, Phe, and the stop codons exhibited values below 1.2, suggesting minimal codon bias for these amino acids.

The mitochondrial genomes of *D. huoshanense*, *D. nobile*, *D. flexicaule*, and *D. officinale* comprise 17 coding sequences (CDs), including essential genes such as *atp4, atp6*, and *cox1*. Notably, *D. nobile* uniquely possesses the *cox2* gene. The GC content across these species ranges from 35.64% to 51.34%, indicating minor disparities in genomic regions (**Supplementary Figure S6**). The average effective number of codons (ENC) exceeding 35 across the species suggests a weak codon usage bias. Neutrality plots illustrating the balance between GC content at different codon positions show minimal slope, indicating that mutation pressure may play a subdued role in shaping codon usage (**Supplementary Figure S7**).

### 3.3 Ka/Ks analysis to determine evolutionary pressures

We computed the non-synonymous to synonymous mutation ratio (Ka/Ks) for 25 common protein-coding genes (PCGs) across the *Dendrobium* species to identify evolutionary adaptations in response to environmental pressures (**Supplementary Figure S8**, **Supplementary Table S2.15**). Notably, genes such as *ccmFC*, *matR*, *mttB*, *nad7*, *rps1*, *rps2*, and *rps10* exhibited higher Ka/Ks values, suggesting these genes may be under positive selection. In particular, the *rps10* gene displayed a Ka/Ks ratio of 2.196, indicating significant evolutionary divergence (P<0.05). This analysis underscores the adaptive responses of mitochondrial genomes to environmental factors, highlighting the potential evolutionary dynamics within the *Dendrobium* genus.

### 3.4 Analysis of repeat sequences in mitochondrial DNA across four *Dendrobium* species

**Figure 4:**
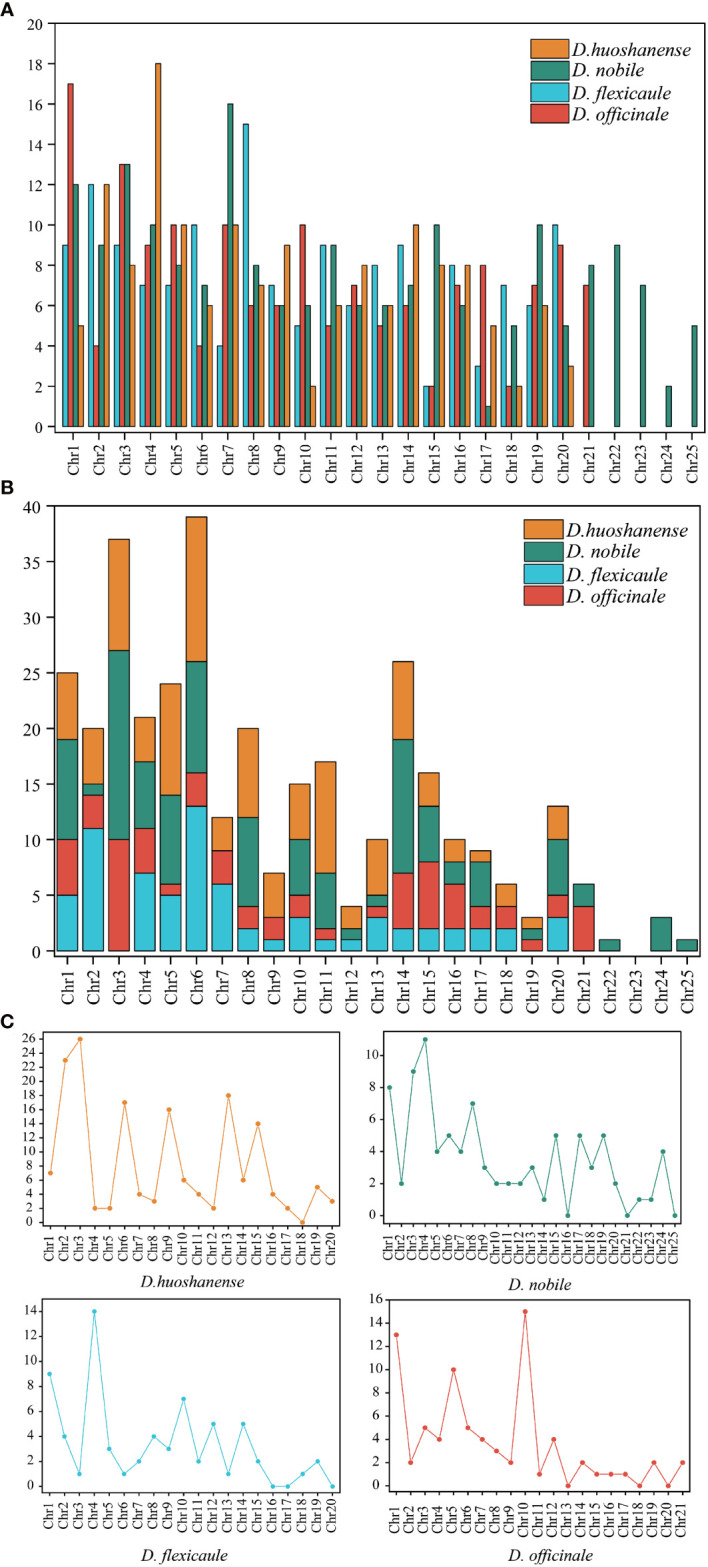
Analysis of repetitive sequences in mitogenomes of four *Dendrobium* species. **(A)** Simple sequence repeats types. **(B)** Tandem repeats types. **(C)**Dispersed repeats types (≥30 bp).

Mitochondrial DNA (mtDNA) in flowering plants, including the *Dendrobium* genus, typically exhibits high complexity due to the substantial presence of repeated sequences. Our investigation into four *Dendrobium* species revealed varied repeat sequences within their mtDNA, detailed comprehensively in **Table 3** and illustrated in **Figure 4**. Across these species, a total of 647 simple sequence repeats (SSRs) were cataloged. The distribution was as follows: *D. officinale* possessed 154 SSRs, *D. flexicaule* had 153, *D. huoshanense* recorded 149, and *D. nobile* had the highest with 191 SSRs. Tetranucleotide repeats were the predominant SSR type in all four species’ mtDNAs, with percentages of 29.87% in *D. officinale*, 30.07% in *D. flexicaule*, 28.19% in *D. huoshanense*, and 34.03% in *D. nobile*. Conversely, hexanucleotide repeats were significantly less common, with no occurrences noted in *D. huoshanense*’s mtDNA.

**Table 3:** Number of repeat sequences from the *Dendrobium* species.

	*D. huoshanense*	*D. nobile*	*D. flexicaule*	*D. officinale*
SSRs	149	191	153	154
Tandem repeats	104	107	71	63
Dispersed repeats (>29 bp)	164	89	66	77

Furthermore, the mtDNA of these species contained a total of 345 tandem repeats, distributed as follows: 63 in *D. officinale*, 71 in *D. flexicaule*, 104 in *D. huoshanense*, and 107 in *D. nobile*. Additionally, 396 dispersed repeats were identified. Of these, forward repeats constituted 76.52% of all dispersed repeats across the mtDNA of the four species, while palindromic repeats accounted for 23.23%. *D. huoshanense*’s mtDNA featured a single 30-bp-long reverse repeat, representing just 0.25% of the total repeat content. These repeat elements were primarily located in the intergenic spacer regions between *trnH* and *trnL*.

Notably, certain chromosomes within the mitochondrial genomes of these species did not harbor any dispersed repeats, indicating unique structural characteristics within their genomic architecture. These chromosomes include *D. officinale* (chromosomes 13, 18, and 20), *D. flexicaule* (chromosomes 16, 17, and 20), *D. huoshanense* (chromosome 18), and *D. nobile* (chromosomes 16, 21, and 25).

### 3.5 Analysis of intracellular gene transfer in *Dendrobium* species

**Figure 5:**
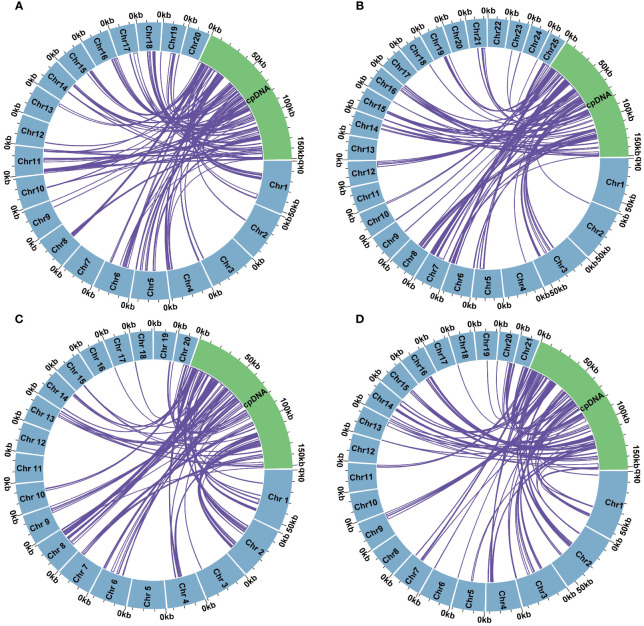
Intracellular genome transfer in *Dendrobium huoshanense* **(A)**, *Dendrobium nobile* **(B)**, *Dendrobium flexicaule* **(C)**, *Dendrobium officinale* **(D)**. Mitogenomes are shown in blue, chloroplast genomes are shown in green, and the purple arcs within the circular ring represent homologous segments between mitochondrial genome and chloroplast genome.

Global alignment of the organelle genomes revealed heterogeneously distributed homologous regions within certain areas of the plastomes (**Supplementary Table S2.16**). Our analysis highlighted considerable variations in the lengths of sequences transferred from the plastomes to the mtDNAs across the four species: *D. nobile* (91,810 bp), *D. flexicaule* (93,329 bp), *D. huoshanense* (103,176 bp), and *D. officinale* (82,308 bp) (**Figure 5**). These segments, integrated due to active recombination and rearrangement, contributed to a total of 173 complete genes across the mitochondrial genomes, encompassing 115 protein-coding genes (PCGs) and 58 tRNA-encoding genes. The specific impacts on gene complements were as follows: 48 genes in *D. nobile*, 45 in *D. flexicaule*, 40 in *D. huoshanense*, and 40 in *D. officinale* (**Table 4**).

**Table 4:** Analysis of homologous fragments based on the four *Dendrobium* species .

Species	Homologous fragments/Length (bp)	Percentage	Longest fragment/Length(bp)	Complete genes	
				Protein coding genes	tRNA genes
*D. huoshanense*	116/103,176bp	15.85%	MTPT4/4,601bp	*accD, atpB, atpE, atpH, ccsA, cemA, matK, ndhD, ndhE, petA*, *petN, psaC, psaB, psaF, psbI, psbJ, psaL, psbM, psbT, rbcL, rpl14, rpl22, rps2, rps4, rps18*	*trnC-GCA, trnD-GUC*, *trnE-UUC, trnF-GAA*, *trnH-GUG, trnL-UAG*, *trnM-CAU, trnN-GUU, trnP-UGG, trnQ-UUG, trnR-ACG, trnR-UCU, trnT-UGU, trnW-CCA, trnY-GUA*
*D. nobile*	111/91,810bp	11.88%	MTPT26/9,550bp	*accD, atpA, atpB, atpE, atpH, atpI, ccsA, clpP, ndhD, ndhE, ndhJ, petG, petL, petN, psaA, psaB, psaC, psaI, psbB, psbI, psbJ, psbM, psbT, psbZ, rbcL, rpl23, rpl33, rpoB, rps14, rps18, rps4, ycf3*	*trnD-GUC, trnE-UUC, trnF-GAA, trnM-CAUx2*, *trnG-UCC, trnH-GUG, trnL-UAG*, *trnN-GUU, trnP-UGG, trnQ-UUG, trnR-UCU*, *trnS-GGA, trnT-UGU, trnW-CCA, trnY-GUA*
*D. flexicaule*	103/93,329bp	15.65%	MTPT53/5,031bp	*accD, atpA, atpB, atpE, atpH, atpI, clpP, matK, ndhD, ndhE, ndhJ, ndhK, petA, petG, petL, petN, psaB, psaC, psaI, psbB, psbI, psbJ, psbM, psbT, psbZ, rbcL, rpl23, rpl33, rpoB, rps14, rps18*	*trnD-GUC, trnE-UUC, trnF-GAA, trnM-CAU, trnG-UCC, trnH-GUG, trnI-CAU, trnL-UAG, trnN-GUU, trnP-UGG, trnQ-UUG, trnR-UCU, trnW-CCA, trnY-GUA*
*D. officinale*	110/82,308bp	13.16%	MTPT57, MTPT58/4,492bp	*accD, atpB, atpE, atpH, atpI, ndhA, ndhD, ndhE*, *ndhG, ndhH, ndhJ, petG, petL, petN, psaC, psaI, psbA, psbB, psbI, psbJ, psbM, psbT, rbcL, rpl23, rpl33, rps4, rps18*	*trnD-GUC, trnE-UUC, trnF-GAA, trnH-GUG*, *trnI-CAU, trnL-UAG, trnN-GUU, trnP-UGG*, *trnQ-UUG, trnR-UCU, trnT-UGU, trnW-CCA*, *trnY-GUA*

“x2” represent that the gene was copied twice.

In *D. flexicaule*, the transferred fragments, representing 15.65% of the total transferred DNA, included 45 complete genes (31 PCGs and 14 tRNAs). Notably, the longest homologous fragment identified was MTPT53, measuring 5,031 bp and containing several complete genes such as *psbZ*, *trnG-UCC*, *trnM-CAU*, *rps14*, *psaB*, and *ndhK*, along with the partial gene *psaA*. The shortest sequence, MTPT68, spanned merely 33 bp and constituted an intergenic spacer.

*D. huoshanense* exhibited the most extensive collection of homologous sequences among the species studied, totaling 116 mitochondrial-transferred plastid fragments (MTPTs), which accounted for 15.85% of its mitochondrial genome. The longest sequence in this collection was 4,601 bp, incorporating segments of the incomplete genes *rpoC1* and *rpoB*. *D. officinale* and *D. nobile* followed closely with 110 and 111 homologous fragments, respectively. In *D. officinale*, the most substantial sequences were MTPT57 and MTPT58, which included parts of the incomplete genes *ycf2* and *trnI-CAU*. For *D. nobile*, the longest sequence, MTPT26, spanned 9,550 bp and encompassed eight complete genes: *psbZ*, *trnG-UCC*, *trnM-CAU*, *rps14*, *psaA*, *psaB*, *ycf3*, and *trnS-GGA*.

Interestingly, the *ycf3* gene was exclusive to the mitochondrial PCGs of *D. nobile* and absent in *D. flexicaule*, *D. officinale*, and *D. huoshanense*. The *petL* gene appeared in the PCGs of the homologous segments from *D. flexicaule*, *D. nobile*, and *D. officinale*, while the *petA* gene was specific to the PCGs of *D. flexicaule* and *D. huoshanense*. A comprehensive analysis of the homologous sequences across the four species revealed a significant presence of complete tRNA-encoding genes. Notably, the *trnC-GCA* gene, with an alignment length of 728 bp, was found exclusively in the homologous sequences of *D. huoshanense*’s mtDNA and was absent in the other three species. Additionally, the *trnS-GGA* gene was unique to *D. nobile*, highlighting the specialized function and distribution of these genes within the chloroplast genomes of *D. huoshanense* and *D. nobile*.

We identified several partial pseudogenes in the homologous fragments of the four *Dendrobium* species, including ψ*ndhA*, ψ*ndhD*, ψ*ndhE*, ψ*ndhG*, ψ*ndhH*, ψ*ndhJ*, and ψ*ndhK* (**Table 5**). The lengths of these pseudogenes range from 451 to 5,031 bp. An in-depth analysis revealed that these pseudogenes are predominantly distributed in the large single copy (LSC) and small single copy (SSC) regions of the chloroplast genome. Furthermore, no complete pseudogenes were found in the homologous fragments of *D. nobile*. In contrast, *D. flexicaule* and *D. officinale* exhibited the highest number of pseudogenes, while *D. huoshanense* had the lowest pseudogene count.

**Table 5:** Sequences transferred from the plastomes to the mitogenomes of four *Dendrobium* species.

Species	Chromosome	Length/bp	Homologous sequences	Position	Complete gene	Identity (%)	Region
*Dendrobium huoshanense*	Chr11	29,499	MTPT70	114,027-116,582	ψ*ndh*D , ψ*ndh*E	7.65%	SSC
*Dendrobium nobile*	*-*	*-*	–	–	–	–	–
*Dendrobium flexicaule*	Chr2	47,832	MTPT11	115,731-118,308	ψ*ndh*D, ψ*ndh*E	5.39%	SSC
	Chr2	47,832	MTPT19	49,509-49,959	ψ*ndh*J	0.94%	LSC
	Chr8	30,006	MTPT53	36,185-41,214	ψ*ndh*K	16.77%	LSC
*Dendrobium officinale*	Chr5	30,692	MTPT40	118,184-119,925	ψ*ndh*A, ψ*ndh*H	5.68%	SSC
	Chr16	23,957	MTPT83	115,531-118,070	ψ*ndh*D, ψ*ndh*G	10.69%	SSC

### 3.6 Prediction of RNA editing sites in the four *Dendrobium* species

An extensive analysis of the mitochondrial genomes across four *Dendrobium* species identified a significant number of RNA editing sites, totaling 2,023 across 147 protein-coding genes (PCGs). The distribution of PCGs undergoing RNA editing was fairly consistent, with 36 unique PCGs in *D. flexicaule* and 37 in *D. huoshanense*, *D. nobile*, and *D. officinale*, as detailed in **Supplementary Figure S9**. Notably, the *ccmFN* gene exhibited the highest number of RNA editing events across all four species, with a cumulative total of 168 sites. For *D. officinale* and *D. huoshanense*, the *ccmB* gene experienced significant RNA editing, with each species showing 35 editing events. Similarly, in *D. nobile* and *D. flexicaule*, both the *ccmB* and *mttB* genes demonstrated 35 RNA editing instances each.

An interesting observation was the minimal RNA editing activity in the *atp8* gene, with only a single editing event predicted across all four species. This consistency suggests low variability in RNA editing for this particular gene. RNA editing events predominantly converted cytosine (C) to uridine (U), leading to changes in the amino acid properties of the encoded proteins. Analysis revealed that 23.93%-24.55% of the amino acids retained their hydrophobic characteristics post-editing, while 43.23%-44.12% of amino acids transitioned from hydrophilic to hydrophobic properties. Leucine was the most common amino acid resulting from RNA editing, affecting 178-183 positions, followed by phenylalanine alterations at 113-116 sites. Some RNA editing events even resulted in the transformation of regular amino acids into stop codons, specifically UAA and UGA.

### 3.7 Analysis of collinearity in the four *Dendrobium* species

**Figure 6:**
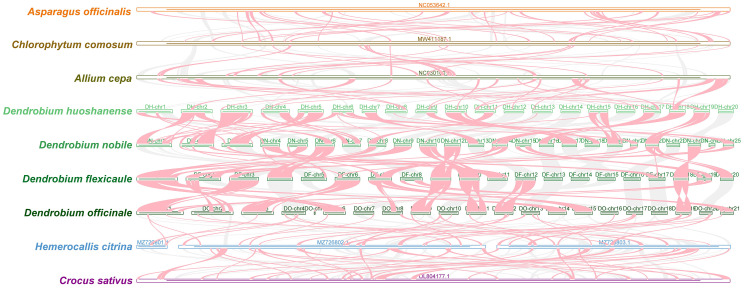
Comparison and analysis of collinearity in four *Dendrobium* species. Perform collinearity analysis between five species and four *Dendrobium* species on the left. Each contig in the figure represents a chromosome. The regions of red arcs suggest inversion areas. The gray regions suggest homologous areas.

The collinearity analysis of mitochondrial genomes within the four *Dendrobium* species used BLASTN to identify conserved homologous sequences. These sequences, considered conserved collinear blocks, were included in the analysis if it exceeded 500 bp and met a size threshold of 0.5 kb. The results indicated an increase in the number of homologous blocks across the four species (**Figure 6**), although these blocks were shorter compared to those in reference species. Notably, a total of 65 homologous collinear blocks ≥7,000 bp were found among the four *Dendrobium* species. The longest block (24.11 kb) was between chromosome 9 of *D. nobile* and chromosome 1 of *D. flexicaule*, while the shortest block (7.15 kb) was identified between chromosome 17 of *D. nobile* and chromosome 15 of *D. flexicaule*. *D. flexicaule* and *D. nobile* were grouped in the same clade in the phylogenetic tree. Additionally, the longest collinear block between *D. huoshanense* and *D. nobile* was found on chromosome 4 of *D. huoshanense* and chromosome 22 of *D. nobile*, measuring 19.93 kb, while the shortest was observed on chromosome 2 of *D. huoshanense* and chromosome 6 of *D. nobile*, measuring 7.15 kb. *D. nobile* and *D. huoshanense* are closely related species.

The presence of blank areas within these plots highlighted notable variations and distinctions in mitochondrial genome homology when compared to other species. The results suggested a divergent order in the arrangement of collinear blocks among Asparagales, indicating significant gene rearrangements within the *Dendrobium* species. The shorter lengths of these collinear blocks across the mitochondrial genomes underscore a lower conservation of sequence order and frequent gene recombination among the mitochondrial DNA of these and other Asparagales species. This complex genetic architecture may reflect adaptive evolutionary processes in response to environmental stresses, influencing the mitogenomic stability and evolution in the *Dendrobium* genus.

### 3.8 Phylogenetic analysis by comparing PCGs of different species

**Figure 7:**
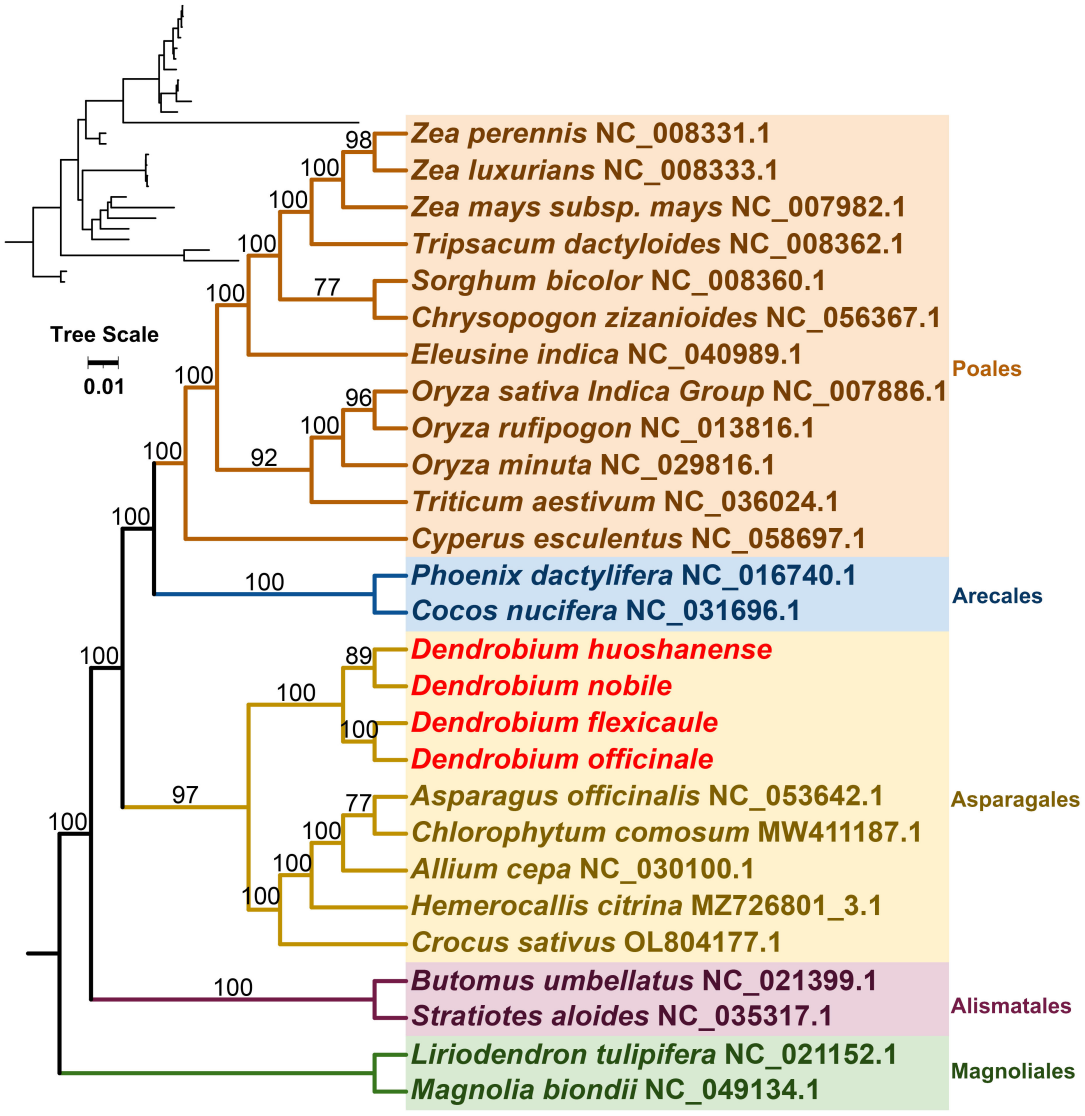
The 21 conserved DNA sequences of protein-coding genes (PCGs) in complete mitogenomes required to construct a phylogenetic tree by selecting 27 species of five orders (Polaes, Arecales, Asparagales, Alismatales, and Magnoliales) in angiosperms. Tree scale was 0.01. The number on each node is the bootstrap values. *Magnolia biondii* and *Liriodendron tulipifera* were selected as outgroups. The accession numbers of the sequences in each species are indicated in the map.

Mitochondrial genomes from 27 species across five orders of angiosperms were constructed phylogenetic trees in this study (**Figure 7**). The phylogenetic tree was based on the mitogenomic sequences of 21 conserved protein-coding genes (PCGs), including *atp1*, *atp4*, *atp6*, *ccmB*, *ccmC*, *ccmFC*, *ccmFN*, *cob*, *cox1*, *cox2*, *cox3*, *matR*, *nad1*, *nad2*, *nad3*, *nad4*, *nad5*, *nad6*, *nad7*, *nad9*, and *rps12*. Two mitochondrial genomes from the Magnoliales were used as the outgroup. The resulting phylogenetic tree had a topological structure consistent with the classification of the angiosperm phylogeny group, indicating its alignment with the latest classification systems. Among the species analyzed, the four *Dendrobium* species belonged to the Asparagales order within the Orchidaceae family. Within this group, *D. nobile* and *D. huoshanense* were identified as closely related sister species, while *D. officinale* and *D. flexicaule* were also closely related.

## 4 Discussion

### 4.1 Features variations in the mitochondrial genome and chloroplast genome structure

Plant mitochondrial genomes are known for their significant variability in size, ranging from several hundred kilobases to several megabases (Skippington et al., 2015; Jackman et al., 2020; Sullivan et al., 2020; Bi et al., 2023; Zhou et al., 2024). The elongation of mitochondrial genomes in seed plants primarily arises from non-coding content (Alverson et al., 2010; Dong et al., 2018; Bi et al., 2024), including duplicated regions, fragments from plastid genomes acquired through intracellular gene transfer (IGT), and foreign mitogenomic sequences obtained through horizontal gene transfer (HGT) (Gandini et al., 2019). These non-coding regions, such as duplicated sequences and intergenomic transfer fragments, are the main contributors to this size variation (Kubo et al., 2000). Additionally, the multi-chromosome structure of mitochondrial genomes further contributes to their complexity (Chen T-C. et al., 2020; Zhou et al., 2023). In this study, we successfully assembled the complete mitochondrial genomes of four *Dendrobium* species—*D. flexicaule*, *D. nobile*, *D. huoshanense*, and *D. officinale*—using a hybrid assembly approach that combined Illumina and Nanopore sequencing technologies. We uncovered a common multibranch conformation in the mitochondrial structures of these four *Dendrobium* species, a characteristic feature of the Orchidaceae family. The mitochondrial genome sizes varied substantially, ranging from 596,506 bp in *D. flexicaule* to 772,523 bp in *D. nobile*. Notably, the total length of the *Cymbidium ensifolium* mtDNA (19 chromosomes, 560,647 bp) also exhibited significant variation compared to *D. flexicaule* within the Orchidaceae family (Shen et al., 2024). Similarly, *D. nobile* (25 chromosomes) and *Paphiopedilum micranthum* (26 chromosomes) differ by only one chromosome in their mtDNAs, yet their mitochondrial genome lengths vary significantly, with a difference of 325,155 bp (Yang et al., 2023a). Meanwhile, the mitochondrial genome of *Apostasia shenzhenica*, (Ke et al., 2023) a closely related species to the four *Dendrobium* species, consists of only one circular molecule, highlighting the remarkable diversity in plant mitochondrial genome structure (Petersen et al., 2020). Interestingly, we also found that non-coding regions and gene numbers might cause variation of mitochondrial genome sizes in Orchidaceae through comparing *D. flexicaule*, *D. nobile*, *D. huoshanense*, *D. officinale*, *A. shenzhenica* and *C. ensifolium*. Despite this variation, the GC content across these genomes was incredibly stable, ranging from 43.41% to 43.64%, suggesting a conservation of GC content across angiosperms over evolutionary timescales (Fan et al., 2022; Niu et al., 2022). Unlike the more conserved and structurally compact animal mitochondria and plastomes, plant mitochondrial genomes are characterized by rapid structural evolution and a high degree of rearrangement.

In our study, the mitochondrial genomes of the four *Dendrobium* species exhibited a sparse distribution of genes amidst a plethora of non-coding sequences. Specifically, *D. flexicaule* and *D. officinale* contained 64 genes, *D. nobile* had 68, and *D. huoshanense* had 69. This pattern mirrors observations in other species (Fan et al., 2022; Li et al., 2022; Niu et al., 2022; Yu et al., 2022). For example, non-coding sequences occupied 91.96% of the total mtDNA of *P. deltoides* (Qu et al., 2023). Additionally, the four *Dendrobium* mtDNAs shared 56 unique genes, comprising 36 protein-coding genes (PCGs), three rRNA-encoding genes, and 17 tRNA-encoding genes. Notably, the *rps11* gene and several tRNA-encoding genes were absent (**Supplementary Table S2.10**), a trend also seen in other species such as *Broussonetia* spp. (Lai et al., 2022) and *Abelmoschus esculentus* (Li et al., 2022). Meanwhile, we found that three (chromosome 11, chromosome 20 and chromosome 24) out of 25 chromosomes in the *D. nobile* mtDNA were found to contain no functional genes, which is similar with previous studies (Alverson et al., 2011; Bi et al., 2022).

### 4.2 RNA editing events

RNA editing is a critical post-transcriptional mechanism in plant organelles that modifies RNA nucleotide sequences to ensure the production of functional proteins (Yan et al., 2018; Zhu et al., 2022). These events can influence protein stability and quantity (Jiang et al., 2022). Within mitochondrial genomes, RNA editing is vital for the accurate translation of mitochondrial genes and the proper functioning of mitochondrial proteins. Additionally, RNA editing plays a crucial role in various aspects of plant biology, including normal biosynthesis in mitochondria and chloroplasts, plant adaptability to environmental conditions, and signal transduction (Yan et al., 2018; Wang and Yang, 2020). This process is essential for the stability and activity of mitochondrial and chloroplast proteins. In the studied *Dendrobium* species, we identified 2,023 RNA editing sites in the mitochondrial genomes of four species, predominantly involving cytosine-to-uridine transitions, which are common in plant mitochondrial genomes (Verhage, 2020; Edera and Sanchez-Puerta, 2021). Notably, while the number of RNA editing sites in *D. flexicaule* (501) and *D. nobile* (502) mtDNAs was similar, there was a significant difference compared to *D. officinale* (510) and *D. huoshanense* (510). Further analysis suggested that the RNA editing events in *atp6*, *ccmFN*, *cob*, *mttB*, *nad3*, *nad4*, *nad7*, *nad9*, and *rps19* genes were the main factors contributing to this variation. The highest number of RNA editing sites in the four *Dendrobium* species was found in NADH dehydrogenase and cytochrome c biogenesis genes, which is consistent with observations in *C. ensifolium*, *Ilex metabaptista* (Zhou et al., 2023), and *Bupleurum chinense* (Qiao et al., 2022).

The consistency of RNA editing events across the four *Dendrobium* species underscores their evolutionary conservation and functional necessity (Edera et al., 2018). This pattern emphasizes the importance of RNA editing in fine-tuning mitochondrial gene products, ensuring appropriate protein functions, and maintaining efficient energy production. Further analysis revealed that the *ccmFN* gene had the highest number of RNA editing sites (42 sites), suggesting extensive modifications crucial for its role in the cytochrome c maturation pathway, essential for mitochondrial electron transport and energy metabolism (Unseld et al., 1997; Giegé et al., 2008). Additionally, 35 RNA editing events were observed in the *ccmB* genes of *D. officinale* and *D. huoshanense*, as well as in the *ccmB* and *mttB* genes of *D. nobile* and *D. flexicaule*. These RNA editing events in specific genes might be functionally significant for their corresponding protein products or essential for their proper assembly into functional complexes involved in mitochondrial electron transport and energy production (Mower et al., 2012). In contrast, the *atp8* gene, which encodes a subunit of the ATP synthase complex critical for ATP production during oxidative phosphorylation, had fewer RNA editing events. This suggests that the *atp8* gene’s nucleotide sequence might be highly conserved and functionally well-adapted in these *Dendrobium* species. Analyzing the patterns of RNA editing events provides insights into the molecular mechanisms governing mitochondrial gene expression and function in these orchids. Comparing these patterns with other orchid species and across different plant lineages might also reveal the evolutionary dynamics of mitochondrial gene regulation and its importance in plant adaptation and diversification.

### 4.3 Analysis of repeat structures and simple sequence repeats

The mitochondrial genomes of flowering plants often exhibit a high level of complexity due to the prevalence of repetitive DNA sequences (Guo et al., 2017; Ma et al., 2022). These repeat sequences are divided into two types: tandem and dispersed repeats. Simple sequence repeats (SSRs), a type of tandem repeat, are commonly used as molecular markers for species identification and genetic diversity (Khera et al., 2015; Ma et al., 2017; Li and Ye, 2020; Liu et al., 2022; Tan et al., 2023; Yang et al., 2023b, c). In our analysis of four *Dendrobium* species, we identified a significant number of SSRs within their mitochondrial DNA (mtDNA). Specifically, *D. officinale* had 154 SSRs, *D. flexicaule* had 153 SSRs, *D. huoshanense* had 149 SSRs, and *D. nobile* had the highest number with 191 SSRs. Tetranucleotide repeats were the most abundant type of SSR across these species, consistent with findings in the mitochondrial genomes of *C. ensifolium*, *A. shenzhenica*, and *L. tsingtauense* (Ke et al., 2023; Qu et al., 2024; Shen et al., 2024). This highlights their potential as molecular markers for genetic diversity assessments and species differentiation within the *Dendrobium* genus (Qiu et al., 2010).

Further examination revealed 352 tandem repeats and 396 dispersed repeats across the mtDNA of these species, with forward repeats accounting for over 76% of all dispersed repeats. These repetitive elements are significant not only for their structural roles but also for facilitating frequent recombination events within the mitochondrial genomes. Such recombination can lead to genomic rearrangements that may influence gene function and plant adaptation (Cole et al., 2018; Dong et al., 2018; Sullivan et al., 2020).

### 4.4 Codon usage bias patterns and evolution

The study of codon usage bias provides essential insights into the evolutionary adaptations and functional constraints of genomic sequences (Nair et al., 2013; Kannaujiya et al., 2014; Mazumdar et al., 2017). Various factors influence codon preferences, including natural selection, mutation, gene sequence base composition bias, tRNA abundance, GC content, gene length, protein hydrophobicity, and amino acid conservativeness (Bulmer, 1991; Novembre, 2002; Parvathy et al., 2022). Our analysis revealed that preferred codons typically end in A or U, indicating a conservation of nucleotide composition. This pattern aligns with the evolutionary trends observed in other plant mitochondrial genomes (Jin et al., 2019; Tang et al., 2020).

Our analysis of codon usage neutrality indicated a generally weak correlation across the species, supporting the theory that natural selection, rather than mutational pressure, predominantly shapes codon usage in these genomes. The mitochondrial gene *rps3* in *D. flexicaule* had the lowest ENC values, while the *atp6* gene’s ENC value was below 35 in all four species. Beyond these two genes, the ENC values of mitochondrial genes in the four *Dendrobium* species ranged from 36.61 to 61.00, with most coding genes exhibiting ENC values above 50.00%, indicating a weaker codon preference. Determining the optimal codons can further enhance gene expression efficiency, providing a foundation for future research on the expression regulation of functional genes in *Dendrobium*, as well as predictions of protein structure and function. This research also offers insights into the conservation of *Dendrobium* germplasm resources and artificial cultivation practices. Analyzing codon bias in plant mitochondrial genomes is crucial for studying genetic regulations, phylogenetic relationships, and the evolution of mtDNA.

The Ka/Ks ratio is often used to examine divergence in protein-coding genes (PCGs) under positive or purifying selection (Hurst, 2002). Our study further explored the non-synonymous/synonymous mutation ratios (Ka/Ks) to identify genes under positive selective pressure, indicative of adaptive evolutionary processes (Xie et al., 2019). Faced with environmental pressures and shifts, species may undergo positive selection on specific genes to alter their protein-coding sequences, enhancing their adaptability to new environments and stress responses (Pimpinelli and Piacentini, 2020). Genes with high Ka/Ks ratios might be involved in essential biological processes related to environmental adaptation (Zhang et al., 2016). For example, the Ka/Ks values for the *ccmFC*, *matR*, *mttB*, and *rps2* genes in the four *Dendrobium* mtDNAs were all greater than one, suggesting that these genes could be closely tied to energy metabolism, redox reactions, or other adaptive processes. The notably high Ka/Ks ratio observed for the *rps10* gene hints at its critical role in species’ adaptive evolution, possibly due to its involvement in key cellular processes or environmental responses. Elevated Ka/Ks ratios might also suggest that these genes help maintain genetic diversity among species (Zhong et al., 2023). By subjecting these genes to positive selection, species could bolster their adaptive capabilities and resilience, enhancing their resistance to various environmental pressures (Bijlsma and Loeschcke, 2012). This analysis highlights the importance of mitochondrial genomes in the adaptive evolution of *Dendrobium* species, facilitating their survival and diversification through modifications in key functional genes.

### 4.5 Gene transfer between the mitochondrial and chloroplast genomes

In plant cells, there is frequent and significant transfer of genetic material between chloroplast and mitochondrial genomes, often resulting in chloroplast DNA sequences comprising 1%-12% of the mitochondrial genome (Wendel et al., 2012). In our study of *Dendrobium* species—*D. huoshanense* (116), *D. nobile* (111), *D. flexicaule* (103), and *D. officinale* (110)—we discovered substantial DNA transfers from chloroplasts to mitochondria, totaling 82,308 bp, 91,810 bp, 93,329 bp, and 103,176 bp, respectively. These transfers constitute a notable proportion of the mitochondrial genome—11.88%, 15.65%, 13.16%, and 15.85%, respectively—highlighting significant variation in the extent of gene transfer among species (Adams et al., 2002). Although the length of these transfer fragments was extremely large compared to *C. ensifolium* (38,163 bp), the number of sequences transferred from the plastomes to the mtDNAs was similar (Shen et al., 2024). Additionally, the ratio of transfer fragments to whole mtDNA length was consistent with previous studies (Yang et al., 2023a).

This influx of genetic material is largely attributable to the integration of repetitive and homologous sequences, which are pivotal in the evolutionary expansion of mitochondrial genomes. Such integration events have occurred gradually over evolutionary timescales, incorporating not just coding regions but also often leading to the formation of non-functional pseudogenes due to recombination events (Richardson et al., 2013). Our analysis reveals that some chloroplast genes become pseudogenes upon integration into the mitochondrial genome. These pseudogenes accounted for 0.94%-16.77% of the mtDNAs in three *Dendrobium* species (*D. huoshanense*, *D. flexicaule*, and *D. officinale*) (**Table 5**) and included NADH dehydrogenase genes. In contrast, the sequence transferred from the plastome to the mtDNA on chromosome 8 of the *D. flexicaule* mtDNA was the largest in length, at 5,031 bp. This result indicates that these transfer fragments undergo multiple rounds of recombination (Yang et al., 2023). Interestingly, shareable *ndhD*, *ndhG*, *ndhH*, *ndhJ*, and *ndhK* genes have been lost in the three *Dendrobium* plastomes, but only a pseudo copy of the shareable ψ*ndhD* gene was identified in the three *Dendrobium* mtDNAs. Published studies have indicated that the loss of NADH genes in some plant plastomes results in nonfunctional pseudogenes (Li et al., 2021). However, tRNA genes tend to retain their functionality, suggesting a conservation of transport functions in mitochondrial genomes, a trait especially pronounced in higher plants.

The specific mechanisms and functional consequences of gene transfer between the mitochondrial and chloroplast genomes in *Dendrobium* species remain poorly understood. However, ongoing advancements in whole-genome sequencing are expected to shed light on these processes. This mitogenomic research enhances our understanding of mitochondrial evolution in flowering plants and sets the stage for further exploration of molecular markers and evolutionary relationships within the Orchidaceae family.

### 4.6 Comparison of the chloroplast and mitochondrial genomes of *Dendrobium* species

Comparative genomics has become indispensable in the study of medicinal plants, providing insights into gene differentiation and evolutionary trends (Daniell et al., 2016). By analyzing 21 conserved mitochondrial protein-coding genes (PCGs) across 27 angiosperm species, we constructed a phylogenetic tree that suggests close relationships within *Dendrobium* species. However, discrepancies in phylogenetic alignment between mitochondrial and chloroplast-derived trees highlight potential inconsistencies that warrant further investigation (Zhu et al., 2018).

## 5 Conclusion

In conclusion, this study successfully assembled the complete chloroplast and mitochondrial genomes of four *Dendrobium* species employing a hybrid assembly method. The mitochondrial genomes of these species are characterized by their multi-chromosomal structure, with chromosome size ranging from 20 to 25 and sizes varying between 596,506 bp and 772,523 bp. The observed mitochondrial genome rearrangements among the four *Dendrobium* species suggest the occurrence of homologous recombination within the mitochondrial genomes of this genus. Phylogenetic analyses reveal that *D. nobile* shares a close evolutionary relationship with *D. huoshanense*, whereas *D. officinale* and *D. flexicaule* are more closely aligned. Overall, our findings contribute significantly to the existing knowledge on the diversity, evolutionary dynamics, and potential molecular markers within *Dendrobium* mitochondrial genomes, thereby providing a robust foundation for further studies on the genetic and functional characteristics of these species.

## Data availability statement

The organelle sequences supporting the conclusions of this article are available in GenBank (https://www.ncbi.nlm.nih.gov/) with accession numbers: OR413847-OR413866 (*Dendrobium huoshanense*), OR413867-OR413891(*Dendrobium nobile*), OR413892-OR413911(*Dendrobium flexicaule*), OR413912-OR413932 (*Dendrobium officinale*) (mitochondrial genomes); OR387325 (*Dendrobium huoshanense*), OR387323 (*Dendrobium nobile*), OQ360111 (*Dendrobium flexicaule*), OR387324 (*Dendrobium officinale*) (chloroplast genomes). The raw data has been released through NCBI with the following accession numbers: (1) *Dendrobium huoshanense*: BioProject PRJNA1129804, BioSample SAMN42166854, SRA SRR29650379 (Illumina), SRA SRR29650378 (Nanopore); (2) *Dendrobium nobile*: BioProject PRJNA1129805, BioSample SAMN42166915, SRA SRR29654108 (Illumina), SRA SRR29654107 (Nanopore); (3) *Dendrobium flexicaule*: BioProject PRJNA1129490, BioSample SAMN42166852, SRA SRR29649768 (Illumina), SRA SRR29649767 (Nanopore); (4) *Dendrobium officinale*: BioProject PRJNA1129803, BioSample SAMN42166853, SRA SRR29649991 (Illumina), SRA SRR29649990 (Nanopore).

## Author contributions

LW: Conceptualization, Data curation, Methodology, Software, Writing – original draft. XL: Writing – review & editing. YW: Investigation, Visualization, Writing – review & editing. XM: Writing – review & editing. JQ: Software, Validation, Writing – review & editing. YZ: Software, Validation, Writing – review & editing.
