# Supplementary material for: Comparative analysis of the mitochondrial genomes of four Dendrobium species (Orchidaceae) reveals heterogeneity in structure, synteny, intercellular gene transfer, and RNA editing
Source: Front Plant Sci. 2024 Jul 30;15:1429545. doi: 10.3389/fpls.2024.1429545 (PMC11319272; doi:10.3389/fpls.2024.1429545)

**Additional File 1. Supplementary Figures**

**(Figures S1-S9)**

# Comparative analysis of the mitochondrial genomes of four *Dendrobium* species (Orchidaceae) reveals heterogeneity in structure, synteny, intercellular gene transfer, and RNA editing

**Figure S1.** Map of coverage depth of Illumina sequencing of *Dendrobium nobile* mitogenome

**
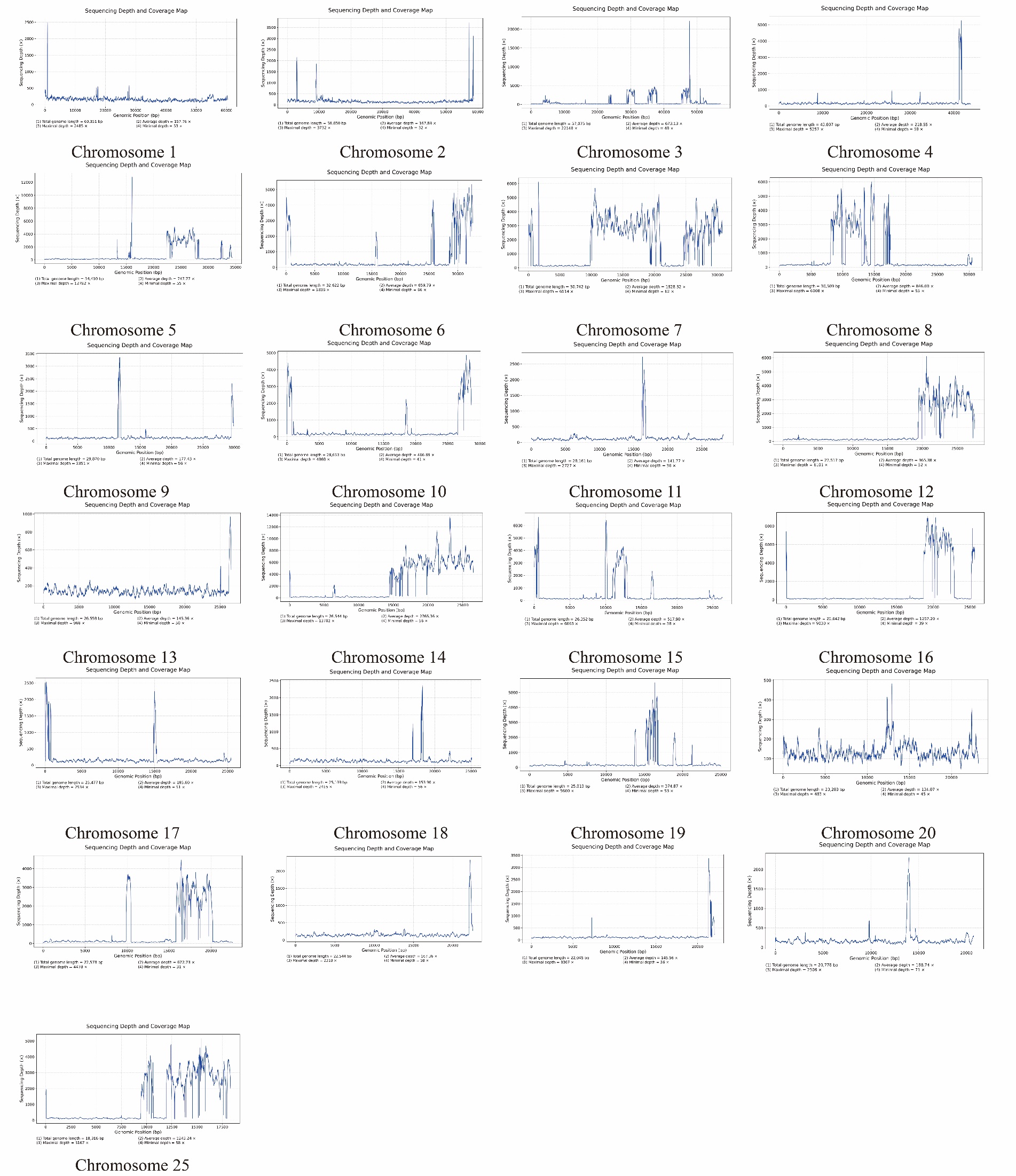
**

**Figure S2.** Sketches of mitochondrial genome of *D. huoshanense* (A), *D. flexicaule* (B) and *D. offcinale* (C)

**
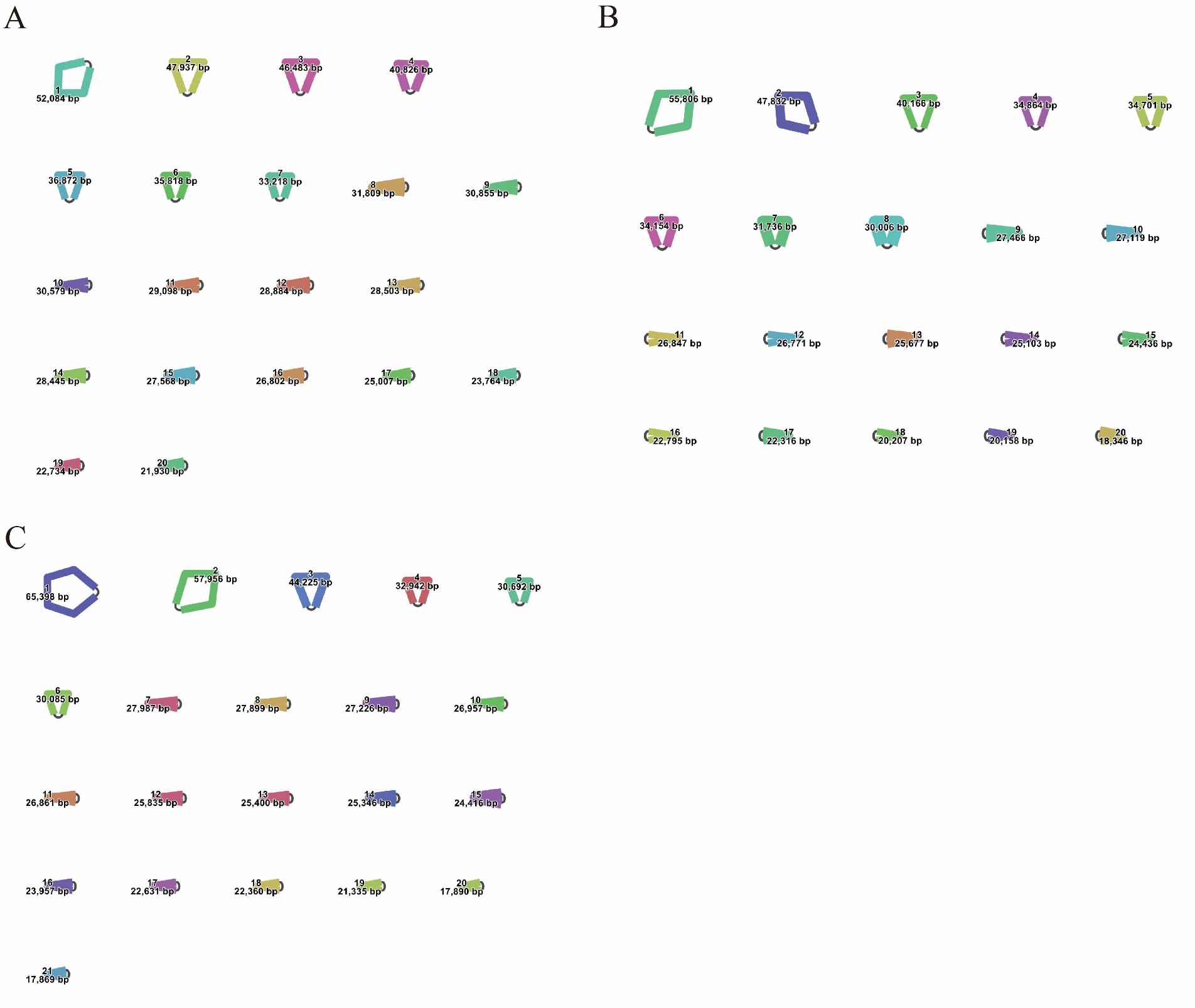
**

**Figure S3.** Mitogenome maps of *D. huoshanense* (A), *D. flexicaule* (B) and *D. officinale* (C)

**
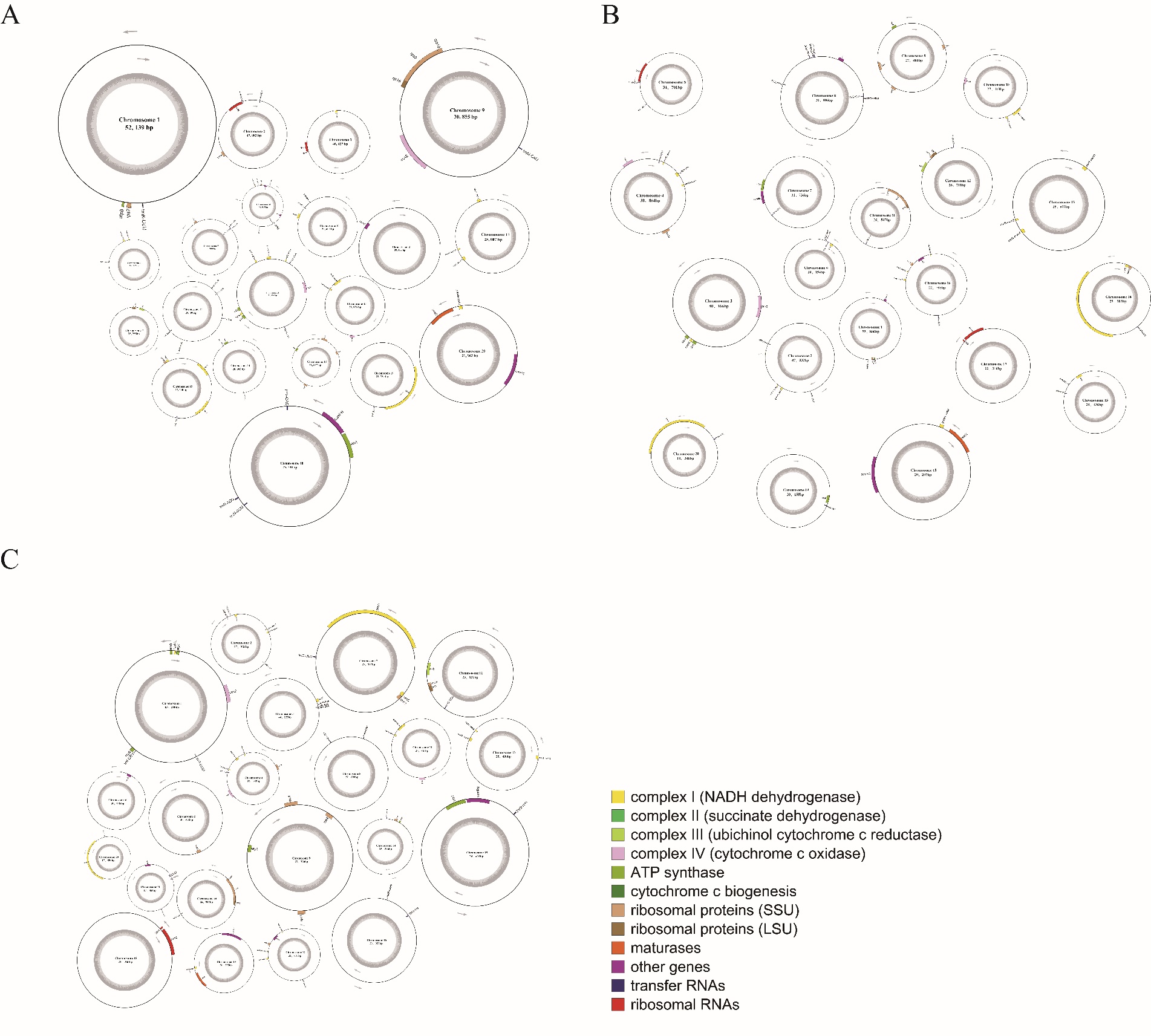
**

**Figure S4.** Complete chloroplast genomes of *Dendrobium huoshanense* (A), *Dendrobium nobile* (B), *Dendrobium flexicaule* (C), *Dendrobium officinale* (D). Note: The red and green arcs lines shown gene migration.

**
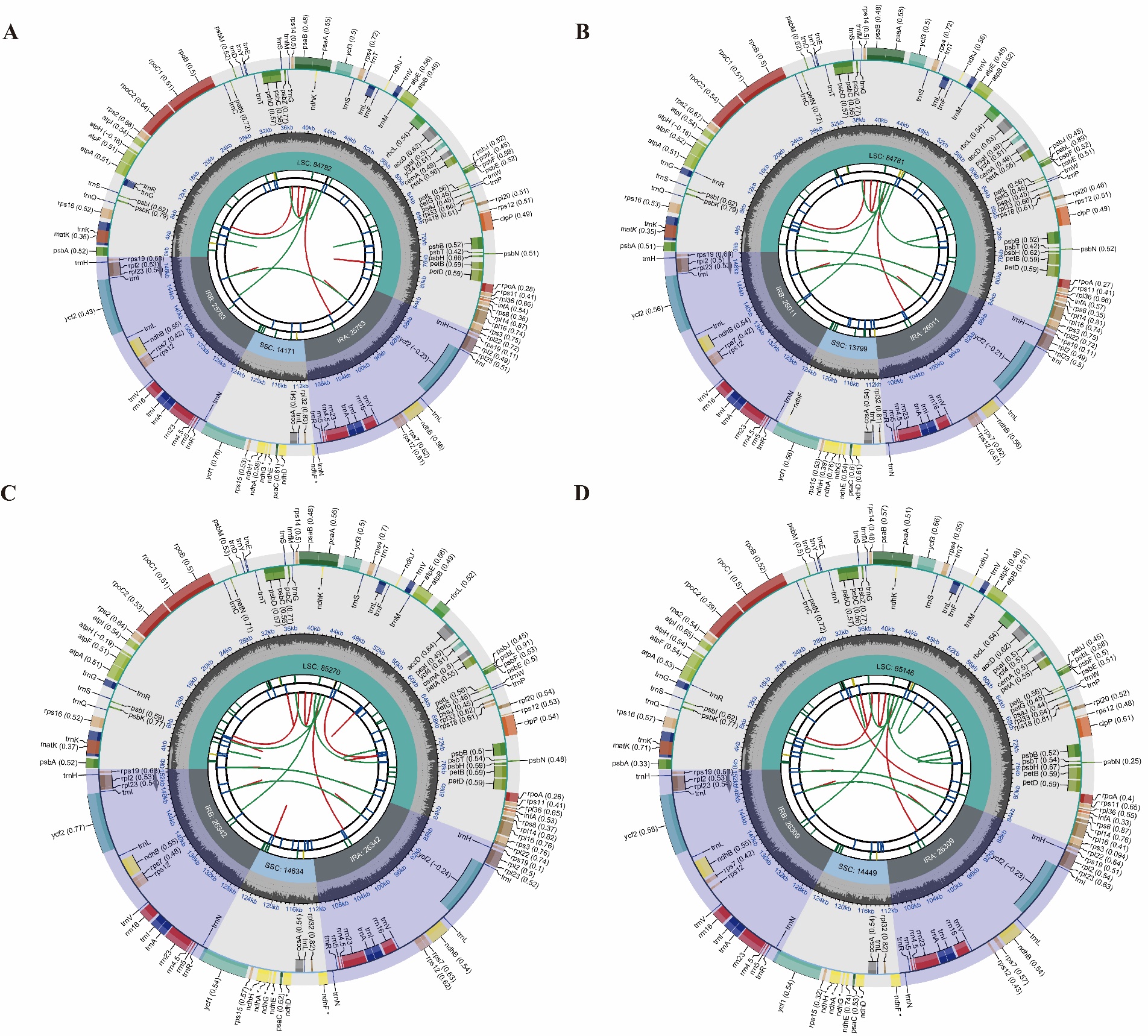
**

**Figure S5.** Visualization alignments of chloroplast genome among four *Dendrobium* species


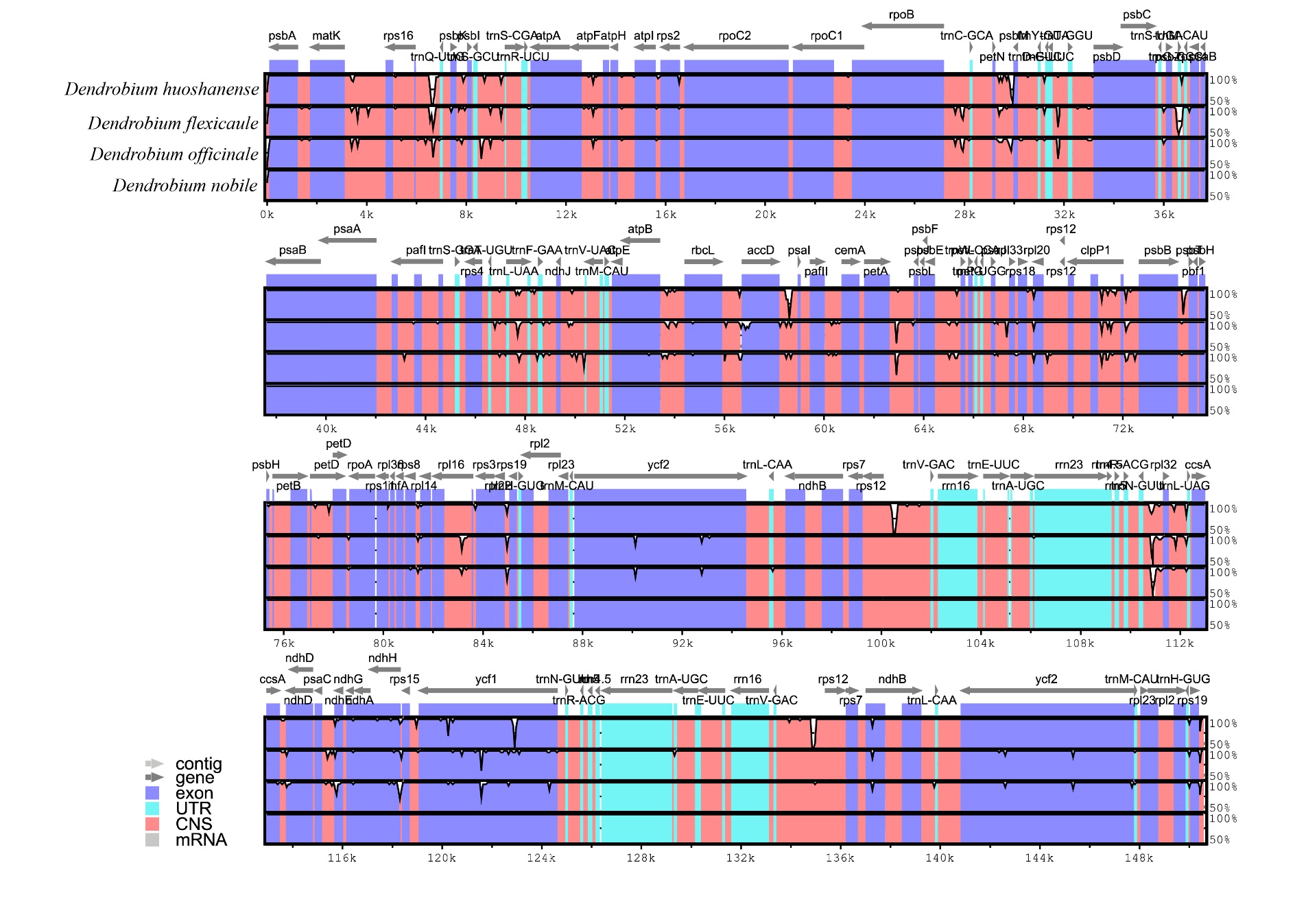


**Figure S6.** GC content of CDs from different positions of the four *Dendrobium* species mtDNA. Note: different colors represent value size in the mtDNA. The blue indicated that *cox*2 was no identified in the three *Dendrobium* species (*D. officinale, D. flexicaule* and *D. huoshanense*).


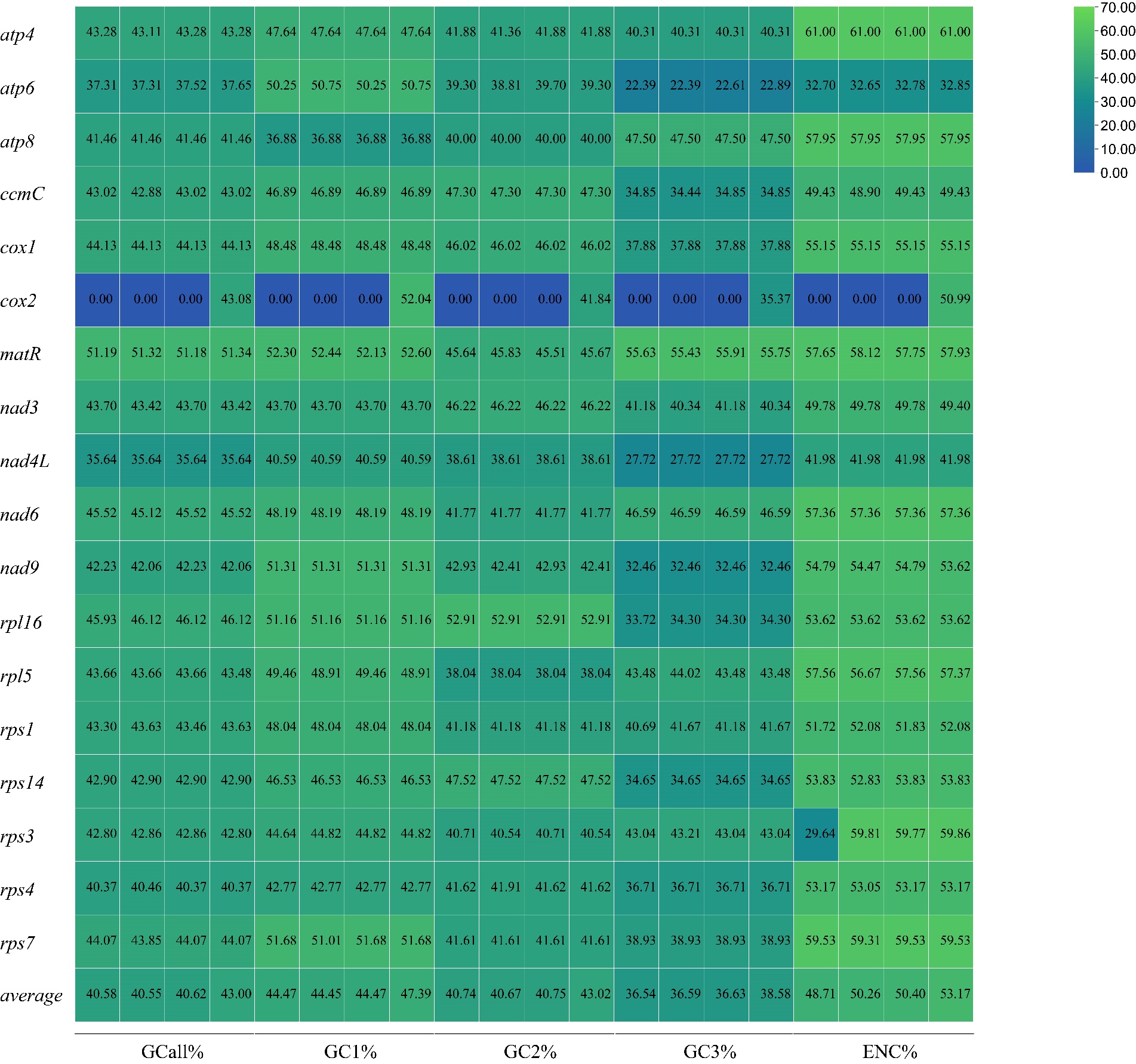


**Figure S7.** Analysis of neutrality from the four *Dendrobium* species (A: *Dendrobium huoshanense*; B: *Dendrobium nobile*; C: *Dendrobium flexicaule*; D: *Dendrobium officinale*). based on the CDs. Note: The different colors represent different CDs.


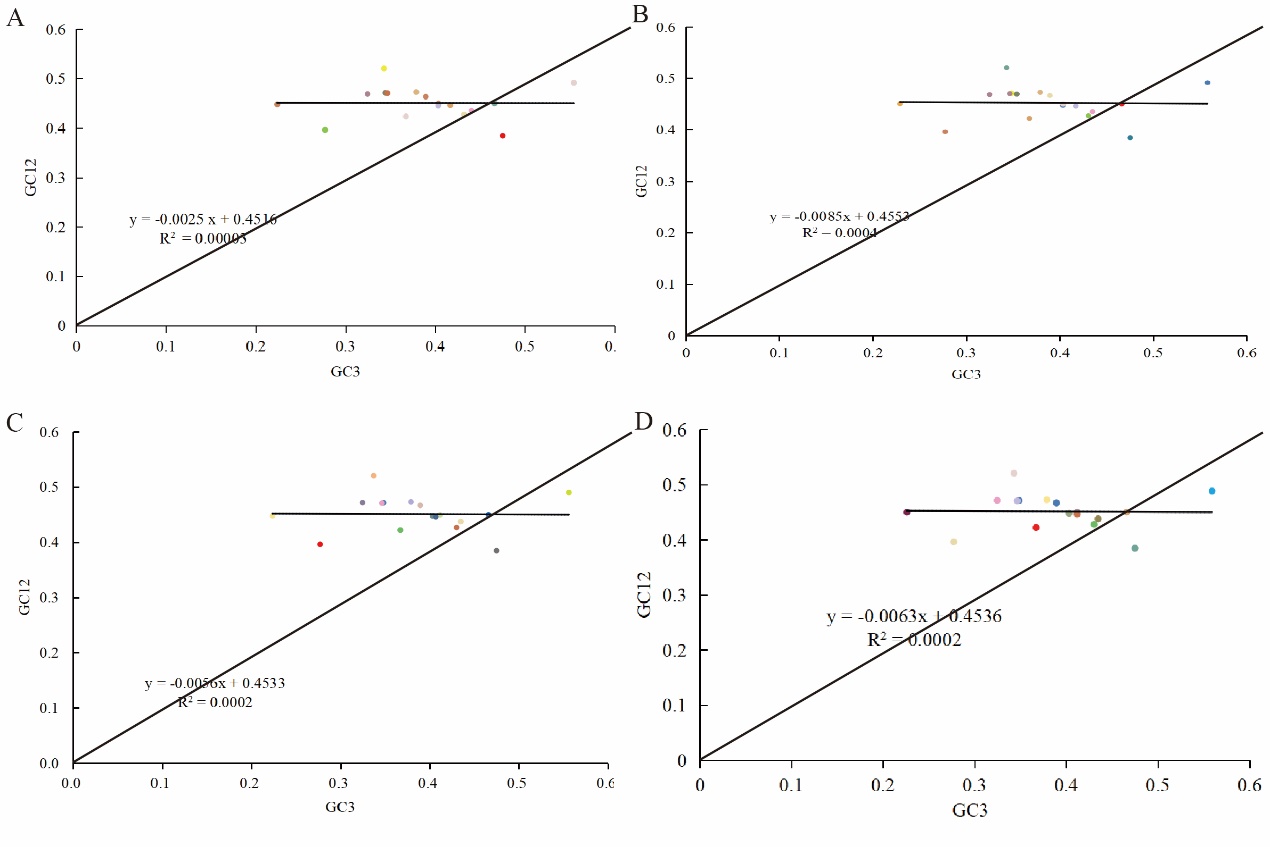


**Figure S8.** Ka/Ks analysis of the 25 shared genes in the four *Dendrobium* species.

**
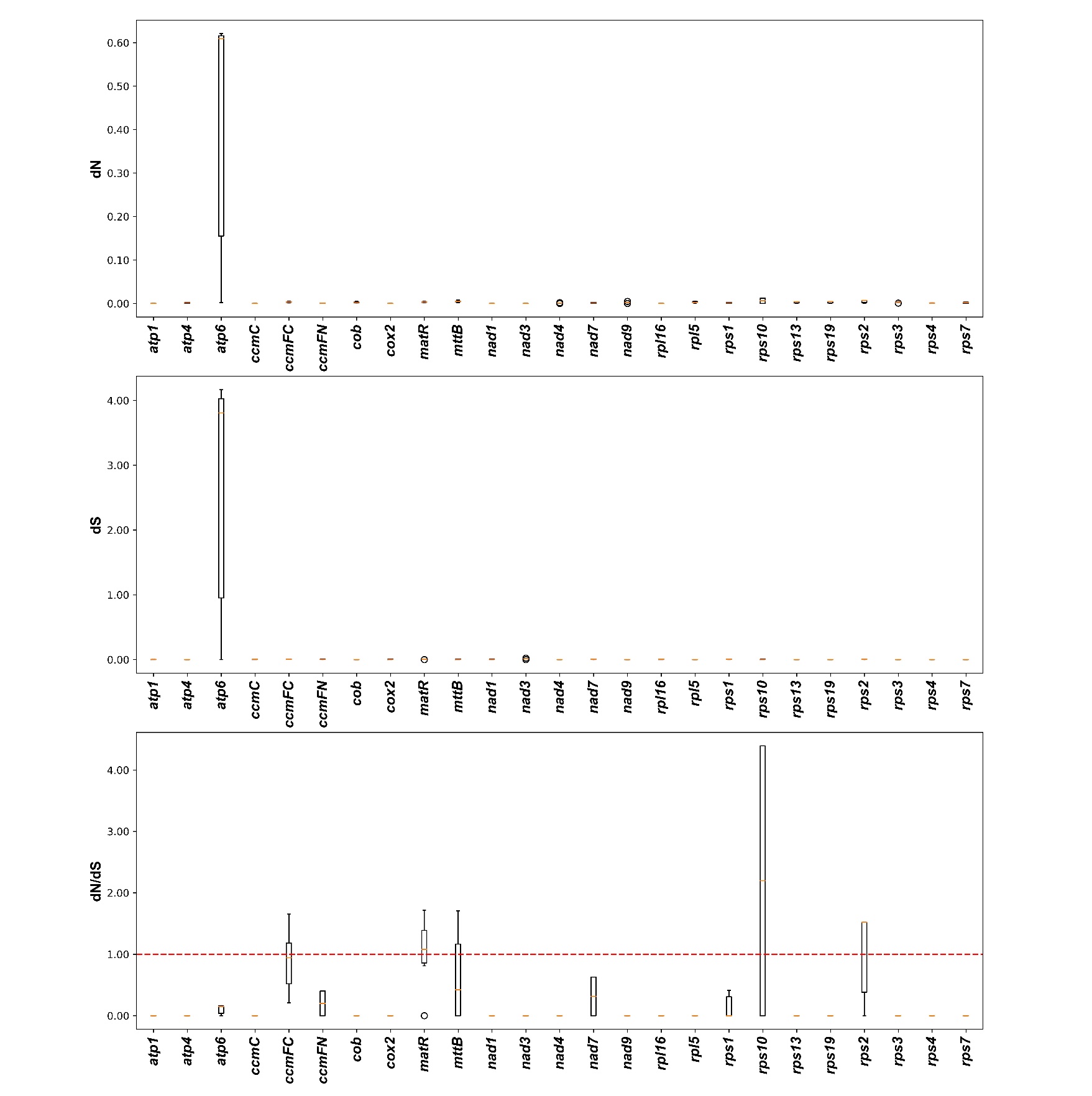
**

**Figure S9.** Prediction of RNA editing sites based on the PCGs. The four *Dendrobium* species was indicated using the different color to filling.


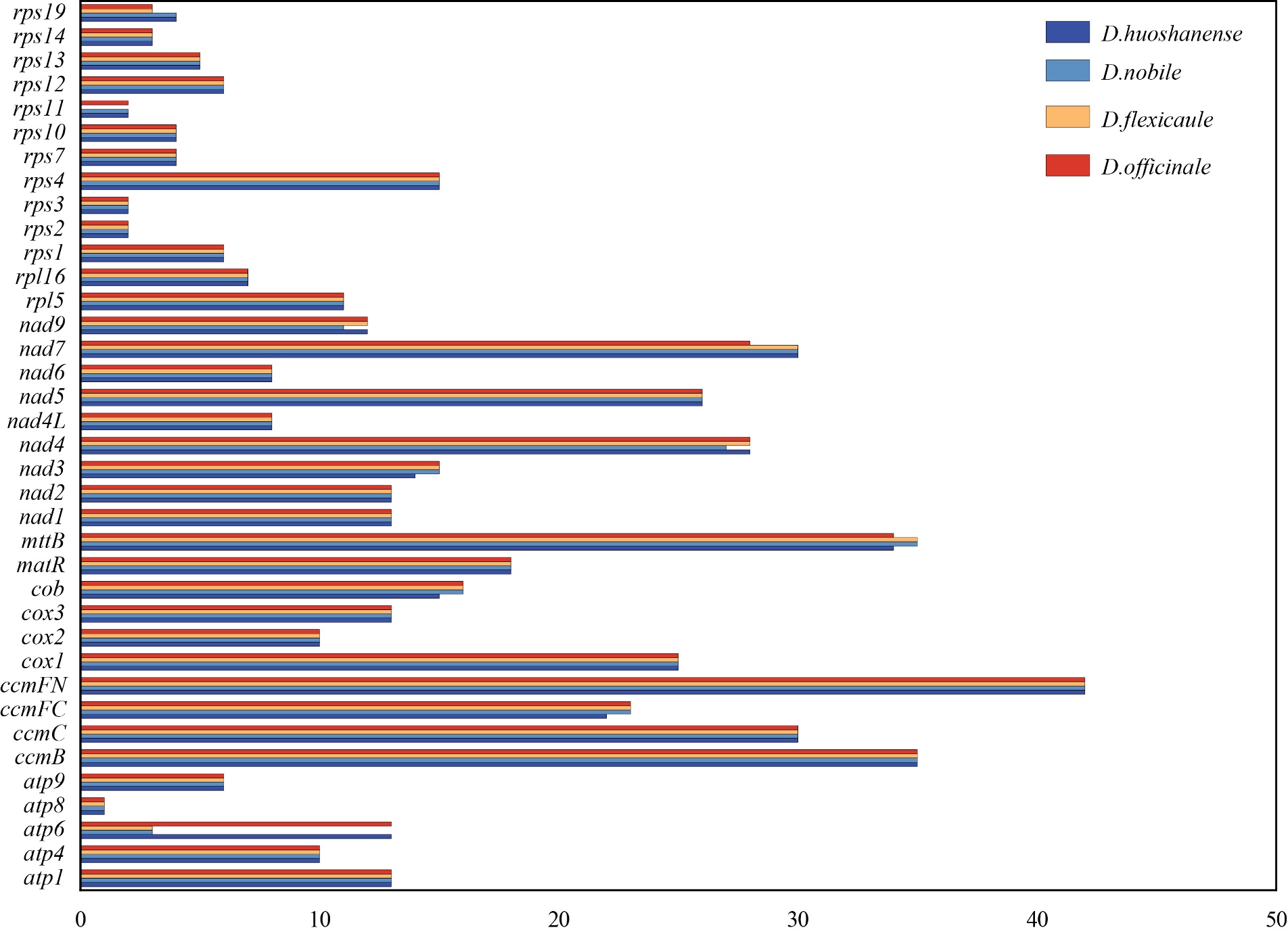

Supplement: Supplementary file 1 [file DataSheet_1.zip › Supplementary Material/Additional File 1.docx]
